# Bacterial secondary metabolites as resistance-modifying adjuvants: microbial origins, molecular mechanisms, and translational relevance

**DOI:** 10.3389/fmicb.2026.1779022

**Published:** 2026-04-10

**Authors:** Victor Uchenna Chigozie, Charles Okechukwu Esimone

**Affiliations:** 1 Department of Pharmaceutical Microbiology and Biotechnology, David Umahi Federal University of Health Sciences, Uburu, Ebonyi, Nigeria; 2 Department of Pharmaceutical Microbiology and Biotechnology, Olivia University, Mukaza, Bujumbura, Burundi; 3 Natural Products Bioprospecting and Translation Research Group (NaPBiTReG), International Institute of Pharmaceutical Research and Innovation (IIPRI), Uburu, Nigeria

**Keywords:** antibiotic adjuvants, antimicrobial resistance (AMR), bacterial secondary metabolites, biofilm disruption, biofilm-associated tolerance, efflux pump inhibition, evolution-resilient therapeutics, microbial evolution

## Abstract

The accelerating crisis of antimicrobial resistance (AMR) necessitates strategies that extend beyond the continual discovery of new conventional antibiotics. Bacterial secondary metabolites, historically valued as sources of antimicrobial scaffolds, are increasingly recognized for their roles as resistance-modifying and anti-virulence agents. This review synthesizes key advances from 2020 to 2025 that reposition bacterial secondary metabolites as integral components of next-phase AMR intervention strategies. We examine their chemical and biosynthetic diversity, ecological functions, and molecular mechanisms of action, including efflux pump inhibition, *β*-lactamase suppression, interference with ribosomal protection, and disruption of biofilms and quorum-sensing networks. Mechanistic and evolutionary analyses are integrated to explain why many metabolites impose higher barriers to resistance development than single-target antibiotics. We further discuss contemporary discovery and optimization pipelines encompassing genome mining, multi-omics approaches, synthetic biology, and AI-assisted structure–activity modeling. Translational considerations are critically evaluated, with emphasis on pharmacokinetic and pharmacodynamic constraints, rational combination therapy design, preclinical validation, and emerging development pipelines. Regulatory, manufacturing, and commercialization challenges are addressed alongside opportunities enabled by nanocarrier delivery systems, microbiome-informed strategies, and personalized medicine. Overall, this review highlights bacterial secondary metabolites as evolution-resilient anti-resistance modalities capable of restoring and extending the efficacy of existing antibiotics, offering a pragmatic and mechanistically grounded path forward in combating AMR.

## 1 Introduction

Antimicrobial resistance (AMR) is a persistent and accelerating threat to global health, undermining the effectiveness of routine medical care, surgery, and infection control worldwide (Chigozie et al., 2025c). Recent global estimates indicate that bacterial antimicrobial resistance was associated with approximately 4.95 million deaths in 2019 and directly attributable to 1.27 million deaths, underscoring both the scale and the urgency of the crisis (Antimicrobial Resistance Collaborators, 2022).

Two linked realities explain why alternative anti-resistance strategies have become a central priority for microbial research and therapeutics. First, the clinical pipeline for truly novel antibiotic classes remains thin relative to the evolving burden of resistance [World Health Organization (WHO), 2025; Brüssow, 2024]; second, pathogens have repeatedly evolved or acquired mechanisms (e.g., target modification, enzymatic inactivation, efflux, biofilm-mediated tolerance) that blunt conventional single-target antibiotics (Nazir et al., 2025; Baweja et al., 2025; Belay et al., 2024). Together, these factors create a pressing need to broaden the conceptual and practical repertoire for combating AMR beyond classical antibiotic discovery.

Bacterial secondary metabolites—the chemically diverse small molecules produced by bacteria through non-ribosomal peptide synthetases (NRPS), polyketide synthases (PKS), ribosomally synthesized and post-translationally modified peptides (RiPPs), terpenoids, alkaloids, and other classes—have traditionally provided many lead structures for antibiotics (Dinglasan et al., 2025; Malfent et al., 2024; Iqbal et al., 2023). However, their functions go far beyond just killing or stopping bacteria. In natural environments, these metabolites are involved in inter-microbial competition, signaling, influencing host responses, and shaping microbial niches. When viewed through the lens of AMR, several of these functions become especially important: directly inhibiting resistance mechanisms (such as efflux pump inhibitors), disrupting protective community behaviors (like biofilms and quorum sensing), modulating host immunity to aid pathogen clearance, and enhancing the effectiveness of existing antibiotics (Azeem et al., 2025; Hetta et al., 2024; Zhang M. et al., 2023; Gaurav et al., 2023). Together, these modes of action suggest that bacterial secondary metabolites could serve as new anti-resistance strategies to restore susceptibility in multidrug-resistant (MDR) pathogens. The shift in perspective—from seeing metabolites only as antibiotics to considering them as supportive agents or tools that alter treatment modalities—is a key aspect of the framework presented in this review.

A range of technological advances has driven progress in discovery and mechanistic understanding. Integrated multi-omics (genomics, transcriptomics, proteomics) combined with untargeted metabolomics and molecular networking now speed up the identification of new metabolites and the mapping of their biosynthetic gene clusters (BGCs) (Singh et al., 2025; Singh et al., 2022). These methods not only enable the detection of novel compounds but also allow quick linkage of molecules to their producing organisms and the genetic basis of their biosynthesis—essential for pathway engineering and scalable production (Ouchene et al., 2022). Contemporary genome-mining platforms and BGC annotation tools also reveal that bacterial biosynthetic potential is vastly underexplored, especially among understudied taxa and environmental niches; systematic mining therefore offers a high-yield route to discovering new anti-resistance candidates (Meesil et al., 2023).

Mechanistic insights are rapidly growing for specific metabolite classes and action modes directly relevant to re-sensitization strategies. For instance, comprehensive research into natural efflux pump inhibitors (EPIs) has mapped how small molecules can bind, block, or disrupt major transporter families, restoring intracellular antibiotic levels and effectiveness against MDR strains both *in vitro* and *in vivo*. These advances demonstrate how metabolite-based adjuvants can be justified, refined, and combined with existing antibiotics to overcome resistance (Zhang S. et al., 2023).

Despite this progress, important gaps remain. These gaps include: limited understanding of how metabolites influence resistance evolution, challenges in scaling natural product biosynthesis, variability in bioactivity across physiological contexts, and incomplete mechanistic characterization for many newly identified compounds. Addressing these challenges requires a comprehensive synthesis of emerging mechanistic, evolutionary, and translational evidence—an objective that motivates this review. This work, therefore, provides an integrated examination of bacterial secondary metabolites as next-generation anti-resistance modalities, emphasizing validated molecular mechanisms, evolutionary considerations, discovery platforms, and translational potential.

## 2 Review methodology

This work was conducted as a comprehensive narrative review, designed to synthesize current evidence on bacterial secondary metabolites and their potential as next-generation solutions to antimicrobial resistance. The review adhered to accepted best practices for scholarly narrative synthesis in microbiology, focusing on breadth, mechanistic detail, and critical evaluation rather than systematic quantitative analysis.

Relevant peer-reviewed literature published between 2020 and 2025 was identified through structured searches of major academic databases, including PubMed/MEDLINE, ScienceDirect, ProQuest, and the Frontiers journal platform. Searches utilized both controlled vocabulary and free-text terms covering the thematic scope of this review, such as “bacterial secondary metabolites,” “antimicrobial resistance,” “efflux pump inhibition,” “biofilm disruption,” “natural product adjuvants,” “resistance resensitization,” “biosynthetic gene clusters,” and “metabolite–pathogen interactions.” Boolean operators and field-specific filters were used to expand retrieval while maintaining relevance.

*Inclusion criteria consisted of: (I)*. Primary research articles presenting experimentally validated molecular, biochemical, genomic, or metabolomic findings; *(II)*. Peer-reviewed reviews and meta-analyses providing mechanistic insights or methodological advances; *(III)*. Translational studies demonstrating *in vitro*, *in vivo*, or preclinical relevance of bacterial metabolites as anti-resistance agents.

*Exclusion criteria included:* Grey literature, non-peer-reviewed sources, speculative commentary lacking experimental support, and studies with unverifiable claims. Reference lists of key publications were manually screened to find additional relevant works not captured by database searches. All retrieved information was verified for accuracy, methodological rigor, and consistency with scientific consensus to prevent the inclusion of unverified or outdated claims. This approach ensures the review presents a rigorous, current, and comprehensive synthesis of mechanistic and translational progress in bacterial metabolite research.

## 3 Bacterial secondary metabolites—diversity, biosynthesis, and ecological roles

### 3.1 Chemical and taxonomic diversity of bacterial secondary metabolites

Bacterial secondary metabolites encompass a remarkably broad spectrum of chemical scaffolds, frequently encoded by dedicated biosynthetic gene clusters (BGCs). Common classes include polyketides, non-ribosomal peptides (NRPs), ribosomally synthesized and post-translationally modified peptides (RiPPs), terpenoids, alkaloids, saccharides, and hybrid compounds combining multiple biosynthetic logics. Recent genomics-scale surveys have revealed that the biosynthetic capacity of bacteria remains vastly underexplored. For example, a global ocean-microbiome analysis estimated on the order of ≈ 64,217 BGCs across marine bacteria, comprising more than 60 distinct biosynthetic types (Al-Siyabi et al., 2025).

Similarly, taxonomic groups not traditionally associated with rich natural-product output—such as members of the genus Corynebacterium—have recently been shown to possess unexpectedly high BGC counts and predicted metabolite diversity (Cunningham et al., 2025). This expanding recognition underscores the notion that “natural product–rich” bacteria are not limited to classical producers (e.g., actinomycetes), but may be phylogenetically widespread.

In environmental and host-associated microbiomes (e.g., soil, marine sponges, aquatic sediments, animal guts), bacterial communities harbor thousands of previously uncharacterized BGCs—implying a vast “dark matter” of secondary metabolite chemistry awaiting discovery (Dat et al., 2022; Du et al., 2024).

Thus, these genomics-scale findings are concordant with global assessments of AMR burden and the urgent need for new, complementary anti-resistance strategies: as bacterial pathogens continue to develop resistance to existing drug classes, the uncovering of large, previously hidden chemical libraries provides both novel lead scaffolds and mechanistic diversity that may be exploited to overcome resistance phenotypes (Antimicrobial Resistance Collaborators, 2022).

### 3.2 Biosynthetic gene clusters and biosynthesis machinery

#### 3.2.1 Genetic organization: BGCs and modular enzymes

Secondary metabolite biosynthesis in bacteria is typically organized via biosynthetic gene clusters: contiguous sets of genes encoding enzymes, regulators, transporters, and accessory functions required to build complex molecules. At the genetic level, most complex bacterial secondary metabolites are produced by physically clustered sets of genes (BGCs) encoding multi-domain, modular enzymes such as polyketide synthases (PKSs) and non-ribosomal peptide synthetases (NRPSs). These BGCs often encode large, multi-domain “megasynthetases. These megasynthetases operate as assembly lines: discrete modules select, activate, and condense building blocks (acyl or amino-acyl units), while accessory tailoring enzymes (oxygenases, methyltransferases, glycosyltransferases, halogenases) introduce further chemical complexity. Representative BGC families, typical scaffolds, habitats, and anti-resistance activities are presented in Table 1. The combinatorial potential inherent to module swapping, domain inactivation, or tailoring-enzyme recruitment underlies the enormous structural variability observed even among closely related clusters (Dilshad et al., 2022). For example, NRPS modules generally include, at a minimum, an adenylation (A) domain (for substrate activation), a condensation (C) domain (chain elongation), and a thiolation/peptidyl-carrier (T/PCP) domain; additional optional domains (e.g., epimerization, cyclization, tailoring) further increase structural diversity (Sword et al., 2024).

**Table 1:** Representative BGC families, typical scaffolds, habitats, and anti-resistance activities.

BGC family/class	Typical scaffolds (examples)	Common habitats/sources	Documented/plausible anti-resistance activities	Key citations
NRPS (non-ribosomal peptides)	Lipopeptides, cyclic peptides (e.g., surfactin-like, daptomycin-type scaffolds); siderophore-NRPS hybrids	Soil actinomycetes, *Bacillus*, marine bacteria, host-associated taxa	Membrane-active lipopeptides can disrupt biofilms and increase antibiotic penetration; siderophore hybrids enable Trojan-horse delivery of antibiotics; NRPS scaffolds provide chemical diversity for efflux-avoidant targets.	Beck et al. (2023), Blin et al. (2025)
PKS (Polyketides) & PKS–NRPS hybrids	Macrolides, polyenes, aromatic polyketides, hybrid polyketide–peptides	Classical actinomycetes, marine streptomycetes, sponge symbionts	Polyketide scaffolds can target novel bacterial processes and may provide enzyme inhibitors (e.g., β-lactamase modulators) or synergize with antibiotics by perturbing membrane/energy metabolism.	Beck et al. (2023), Blin et al. (2025)
RiPPs (lantipeptides, thiopeptides, others)	Lantibiotic-like peptides, thiopeptides, lanthipeptides	Gram-positive soil bacteria, some gut commensals	Potent narrow-spectrum activity, target cell-wall biosynthesis or ribosomal functions; potential as adjuvants to displace resistant strains or as scaffolds engineered for altered target specificity.	Beck et al. (2023), Blin et al. (2025)
Terpenes/isoprenoids	Mono-, sesqui, diterpenes (volatile and semi-volatile terpenoids)	Plant-associated bacteria, marine bacteria, and soil microbes	Several terpenes act as efflux pump inhibitors (EPIs) or membrane modulators that restore intracellular antibiotic concentrations; thus, frequently reported as resistance-modifying adjuvants.	Dias et al. (2022)
Alkaloids/phenazines/redox-active small molecules	Phenazines, alkaloid heterocycles	Soil, rhizosphere, marine sediments, host microbiomes	Phenazines and other redox-active metabolites can perturb biofilm physiology, respiratory homeostasis, and generate reactive species that sensitize bacteria to antibiotics; some alkaloids inhibit resistance enzymes in vitro.	SeyedAlinaghi et al. (2025), Zhai et al. (2023)
Siderophores and siderophore BGCs	Catecholate, hydroxamate, mixed-type siderophores; siderophore-antibiotic conjugates (sideromycins)	Soil, aquatic, clinical isolates	Siderophore pathways provide Trojan-horse strategies (siderophore–antibiotic conjugation) to bypass outer-membrane permeability barriers and deliver payloads into MDR Gram-negative pathogens.	Rayner et al. (2023), Miao et al. (2025)
Small signaling molecules/DKPs/quorum-sensing modulators	Diketopiperazines, halogenated furanones, AHL mimics	Marine biofilms, inter-species co-cultures, host microbiomes	Quorum-sensing inhibitors (QSIs) and biofilm dispersal agents reduce virulence and biofilm tolerance, thereby re-sensitizing communities to antibiotics and lowering selective pressure for resistance.	Qin et al. (2023), Zhang M. et al. (2023)

Polyketide biosynthesis similarly relies on modular PKSs featuring ketosynthase (KS), acyltransferase (AT), acyl-carrier protein (ACP) domains, often supplemented by ketoreductase (KR), dehydratase (DH), enoylreductase (ER), and other tailoring domains (Dilshad et al., 2022; Klaus et al., 2021).

This modularity enables enormous structural variability—small changes in module composition or order can yield vastly different chemical entities.

#### 3.2.2 Hidden potential: silent BGCs and novel sources

Despite the abundance of BGCs in bacterial genomes, only a minority are expressed under standard laboratory conditions; many remain “silent” or cryptic (Sedeek et al., 2025). Activating such silent BGCs is a major focus in natural-product research. Recent studies describe novel pipelines combining long-read sequencing (to correctly assemble complex, often GC-rich genomes), computational BGC mining (e.g., via tools such as antiSMASH), and heterologous or synthetic biology expression to unlock production of previously inaccessible metabolites (Campos-Magaña et al., 2025; Foldi et al., 2024).

Contemporary activation strategies fall into several complementary classes: *(I). Culture-based elicitation and co-culture*—exposing producer strains to chemical elicitors, stressing growth conditions, or co-culturing with other microorganisms can induce expression of silent BGCs via interspecies signaling. *(II). Transcriptional refactoring and promoter engineering*—replacing native, tightly regulated promoters with constitutive or inducible promoters (or integrating transcription-factor decoys) can drive expression. *(III)*. *Heterologous expression*—transferring BGCs into well-characterized expression hosts (e.g., Streptomyces chassis, engineered *E. coli* strains, or yeast) overcomes native regulatory constraints and allows scalable production. *(IV)*. *Genome engineering (CRISPR-Cas approaches, ribosome engineering, regulatory knockouts)*—targeted manipulation of pathway regulators or global transcriptional modulators can derepress cryptic clusters. *(V). High-throughput elicitor screening and reporter-guided selection*—small-molecule libraries and biosensor reporters enable systematic identification of activators.

Moreover, environments beyond classical soil and actinomycete niches—including marine ecosystems, extreme environments (e.g., soda lakes), and anaerobic gut microbiota—are increasingly recognized as reservoirs of novel BGCs. For instance, a recent survey of bacteria from an alkaline soda lake revealed multiple BGCs, including for terpenes, alkaloids, and phenazine-like compounds, reinforcing the value of bioprospecting in non-conventional habitats (Bekele et al., 2025).

Importantly, combining computational detection of BGCs (antiSMASH, BiG-SCAPE, others) with experimental activation pipelines has increased the throughput and success rate of natural-product discovery (Murtaza et al., 2025). Thus, through the use of improved genomic tools and expanded ecological sampling, the known biosynthetic potential of bacteria is rapidly growing.

### 3.3 Ecological and functional roles of secondary metabolites

Bacterial secondary metabolites play central roles in natural microbial ecology, mediating competition, communication, and adaptation to diverse environmental pressures. These functions arise from evolutionary selection within complex communities and provide the ecological foundation for many properties later exploited in antimicrobial and anti-resistance applications.

#### 3.3.1 Competitive interactions and chemical defense

In densely populated microbial environments, competition for nutrients and spatial niches drives the production of inhibitory secondary metabolites that suppress rival organisms or limit their colonization. Such metabolites contribute to structuring community composition and maintaining ecological balance (Mevo et al., 2021; Du et al., 2024). Importantly, the selective pressure for effective chemical defense promotes structural and functional diversification, resulting in metabolites that often act through unconventional targets or modes of action distinct from classical antibiotics (Smith et al., 2023; Nazir et al., 2025). This ecological origin helps explain why many secondary metabolites retain activity against resistant organisms.

#### 3.3.2 Signalling, quorum sensing, and community modulation

Beyond direct antagonism, secondary metabolites frequently function as signaling molecules that regulate population-level behaviors, including quorum sensing, biofilm formation, and interspecies interactions (Chigozie et al., 2025a; Armes et al., 2025). Biosynthetic gene cluster expression is commonly linked to population density, environmental cues, or stress signals, ensuring metabolite production is context-dependent and metabolically economical (Zhgun, 2023; Cawood and Ton, 2025). These signaling roles underpin non-lethal modulation of microbial behavior—such as attenuation of virulence or alteration of collective phenotypes—highlighting ecological strategies that minimize direct killing and may reduce selection for resistance (Rajkhowa et al., 2024; Benyamini, 2024).

#### 3.3.3 Environmental adaptation and community dynamics

Secondary metabolites also facilitate adaptation to extreme or specialized environments, including marine systems, hypersaline or alkaline habitats, and the anaerobic gut. In such contexts, metabolites may mitigate oxidative stress, enable nutrient acquisition, deter predation, or mediate interactions within polymicrobial consortia. Metagenomic studies of bacteria from soda lakes and other extreme environments have revealed diverse biosynthetic pathways, including those encoding alkaloids and phenazines, likely linked to environmental resilience (Bekele et al., 2025). Collectively, these adaptive functions emphasize that secondary metabolites evolved to operate within dynamic ecological networks, providing a conceptual basis for their later repurposing in strategies aimed at destabilizing resistant microbial communities.

### 3.4 Implications for anti-resistance therapeutics development

The vast chemical and functional diversity, combined with the modular biosynthetic logic and ecological context of secondary metabolites, offers multiple advantages for developing anti-resistance therapies:

- Structural novelty: Modular biosynthesis enables scaffolds not found in existing antibiotics—increasing the chances of targeting new biological mechanisms or bypassing current resistance methods.
- Functional versatility: Metabolites may serve as inhibitors of resistance factors (such as efflux pumps or enzymes), modulators of microbial community behavior (like biofilms and quorum sensing), or as agents that alter host–microbe interactions (such as immunomodulation or reducing virulence).
- Evolvability and engineering potential: The modular nature of BGCs supports synthetic biology, pathway modification, combinatorial biosynthesis, and heterologous expression, allowing scalable access to natural products and their derivatives.
- Diverse ecological niches as resource reservoirs: Microbiomes from the environment and hosts—often overlooked—contain a rich and largely untapped reservoir of metabolites.

Overall, these features support a focused, mechanistic, and translational review of bacterial secondary metabolites as promising next-generation anti-resistance therapies.

#### 3.4.1 Interpretive notes

1. Purpose and scope. The table is intended as a compact reference linking biosynthetic classes (BGC families) to the mechanistic categories of anti-resistance activity that they most commonly furnish (e.g., efflux inhibition, Trojan-horse delivery, biofilm dispersion, quorum-sensing antagonism, immunomodulation).
2. Evidence level. For several scaffold/activity pairs (e.g., terpenes as EPIs; siderophores as Trojan-horse delivery systems), there is direct experimental evidence and translational precedent.
3. Link to discovery pipelines. The right-hand column (citations) points to review papers and platform resources (antiSMASH, GNPS, multi-omics) that operationalize discovery of the scaffolds listed—this ties the chemical/biological potential to practical discovery and prioritization workflows.

## 4 Molecular mechanisms underlying bacterial secondary metabolites as anti-resistance modalities

This section integrates chemical classes, biosynthetic drivers, ecological rationales, and experimentally validated effects on multidrug-resistant (MDR) bacteria. Where possible, quantified outcomes (e.g., changes in MIC, efflux inhibition percentages, biofilm disruption) and assay details are provided.

### 4.1 Chemical and biosynthetic diversity (NRPS, PKS, RiPPs, terpenoids, alkaloids)

#### 4.1.1 Non-ribosomal peptides (NRPs)

**Figure 1:**
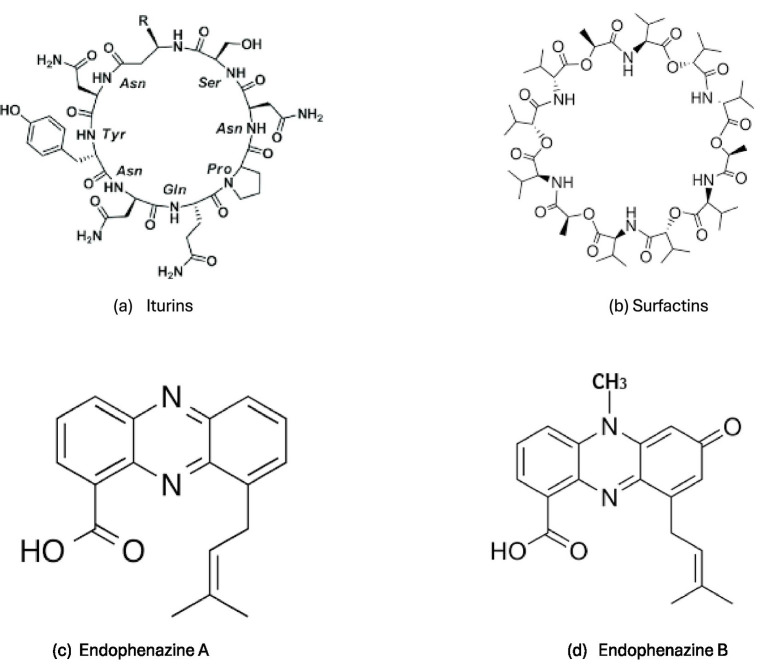
Chemical structures of some *biosynthetically diverse* bacterial metabolites. **(a)** Iturins; **(b)** Surfactins; **(c)** Endophenazine A; **(d)** Endophenazine B. Source: (Yaraguppi, et al., 2023; Kumar et al., 2023; Liu et al., 2022).

*Cyclic Lipopeptides* from *Bacillus* spp.—NRPS biosynthesis yields cyclic amphiphilic lipopeptides (CLPs) such as *iturins* (Figure 1a), *surfactins* (Figure 1b), and *fengycins* which structurally combine peptide rings with fatty acid chains. These amphiphilic metabolites destabilize microbial membranes, influence community behavior, and in some contexts enhance antibiotic uptake or disrupt biofilms (Yaraguppi et al., 2023; Malviya et al., 2020; Lin et al., 2020). CLPs have demonstrated membrane perturbation that can significantly reduce biofilm biomass in MDR pathogens and sensitize them to antibiotics when co-applied *in vitro* (Md Gulzar et al., 2025). Bacillus CLPs can also trigger host immune responses that indirectly affect pathogen resilience. The amphiphilic character, derived from NRPS modularity, is critical to these activities (Markelova and Chumak, 2025; Ding et al., 2025).

Assay details (example): *In vitro* biofilm inhibition assays using crystal violet quantification show that surfactin concentrations as low as 50–100 μg/mL can reduce biofilm biomass in *Staphylococcus aureus* and *Pseudomonas aeruginosa*, with further potentiation of sub-MIC antibiotics. CLPs also alter membrane potential in target cells (e.g., increased propidium iodide uptake), consistent with membrane disruption effects (Markelova and Chumak, 2025). Although CLPs themselves are not typically classified as “efflux pump inhibitors,” their capacity to alter membrane integrity can enable otherwise excluded antibiotics to penetrate resistant cells—effectively lowering MICs and contributing to re-sensitization.

*Polyketides (PKS-Derived)*–Polyketide biosynthesis contributes to macrocyclic structures and aromatic polyketides with complex ring systems. These include phenazine-like heterocycles and unusual macrolides. Polyketide scaffolds are often associated with redox chemistry and electron transfer, which can disrupt respiratory homeostasis in resistant cells and enhance sensitivity to bactericidal drugs (Kumar and Chopra, 2025).

For example, Phenazine derivatives (Figures 1c,d) produced by environmental *Pseudomonas* strains have been shown to interfere with biofilm maturation and to act synergistically with aminoglycosides against *P. aeruginosa* biofilms, reducing viable biomass by >2 logs at combined doses *in vitro* (Demisie et al., 2024). Biofilm inhibition was quantified by CFU enumeration and confocal microscopy, showing increased antibiotic penetration and decreased extracellular matrix. The phenazine compound did not alter MIC in planktonic culture but significantly reduced biofilm tolerance when co-administered with antibiotics (Thalhammer and Newman, 2023).

### 4.2 Ribosomally synthesized and post-translationally modified peptides (RiPPs)

RiPPs encompass diverse peptides, including lasso-peptides and lanthipeptides. These compounds may target ribosomal functions, cell envelope biosynthesis, or specific intracellular processes.

#### 4.2.1 Lariocidin

**Figure 2:**
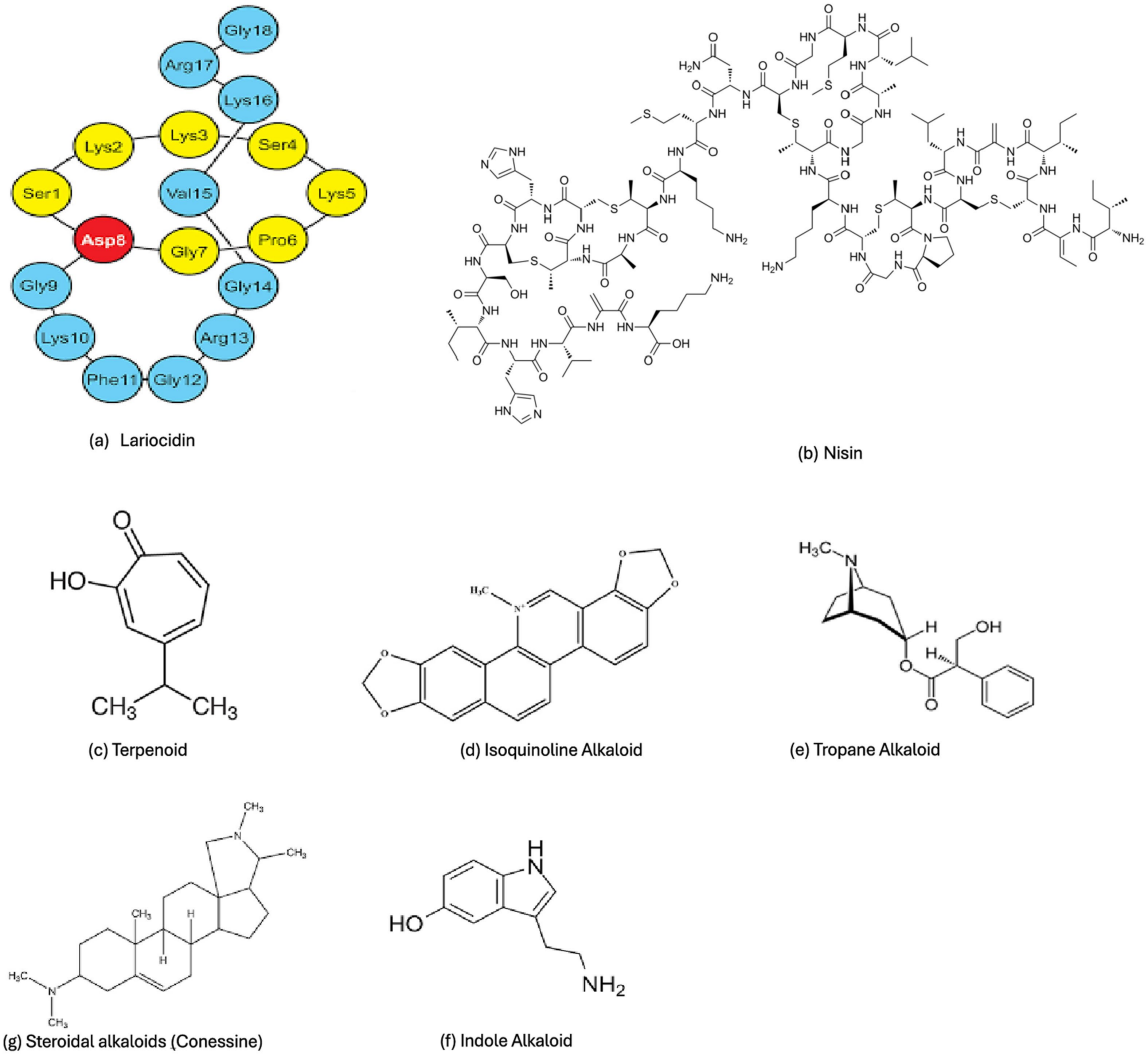
Chemical structures of some ribosomally synthesized and post-translationally modified peptides. **(a)** Lariocidin (a Lasso-peptide); **(b)** Lanthipeptides (Nisin); **(c)** Terpenoid; **(d)** Isoquinoline Alkaloid; **(e)** Tropane Alkaloid; **(f)** Indole Alkaloid; **(g)** Steroidal alkaloids (Conessine).

A newly reported lasso-peptide (Figure 2a) from *Paenibacillus* sp. has been characterized in 2025 as a potent inhibitor of ribosomal translation that retains activity against WHO priority pathogens, including carbapenem-resistant *Klebsiella pneumoniae*, *Acinetobacter baumannii*, and MDR *E. coli* (Jangra et al., 2025; Pandey, 2025). The peptide binds the 30S ribosomal subunit and inhibits translocation by interfering with A-site tRNA accommodation, leading to miscoding and bactericidal effects. Time-kill assays with Lariocidin (Figure 2a) demonstrated >3 log10 reduction in CFU/mL for resistant isolates at concentrations near 2 × intrinsic MICs. Ribosomal binding was confirmed via toe-printing assays and competition with canonical translation inhibitors in vitro (Jangra et al., 2025; Pandey, 2025).

#### 4.2.2 Nisin

Nisin, a prototypical lantibiotic (class I lanthipeptide) produced by *Lactococcus lactis*, (Figure 2b) is among the best-characterized ribosomally synthesized and post-translationally modified peptides and continues to serve as a benchmark for membrane- and cell-wall–targeting antimicrobials with resistance-modifying potential (Chen et al., 2020; Knysh and Martynov, 2023). Mechanistically, nisin exerts its antibacterial activity through high-affinity binding to lipid II, a central precursor in peptidoglycan biosynthesis, thereby simultaneously inhibiting cell wall synthesis and inducing pore formation in the cytoplasmic membrane (Tavares et al., 2023). This dual mechanism distinguishes nisin from classical *β*-lactams or glycopeptides and underlies its rapid bactericidal action. Structural and biophysical studies demonstrate that nisin–lipid II complexation sequesters lipid II from penicillin-binding proteins while facilitating the assembly of transient membrane pores that dissipate membrane potential and ion gradients. These effects culminate in rapid loss of viability, with time-kill assays showing multi-log reductions in CFU within hours against susceptible and moderately resistant Gram-positive pathogens, including *Staphylococcus aureus* and *Enterococcus faecium* (Zheng et al., 2022). Importantly, resistance to nisin emerges slowly and is typically associated with fitness costs, reflecting the evolutionary constraint imposed by targeting an essential and highly conserved cell wall precursor.

Beyond intrinsic activity, nisin has been shown to potentiate conventional antibiotics, particularly β-lactams and glycopeptides, by increasing membrane permeability and weakening cell wall integrity (Wang et al., 2023). This adjuvant-like behavior lowers effective MICs of partner drugs and can partially restore susceptibility in strains exhibiting reduced β-lactam sensitivity. Collectively, nisin exemplifies how lanthipeptides achieve robust antibacterial activity through precise molecular targeting of lipid II coupled to membrane disruption, providing a mechanistically well-defined template for developing next-generation lanthipeptide-based anti-resistance strategies.

#### 4.2.3 Terpenoids

Bacterial terpenoid metabolites (Figure 2c) include volatile and semi-volatile isoprenoids that can interact with membranes and regulatory systems at sub-inhibitory concentrations. Terpenoids have been reported to exert efflux pump inhibition (EPI) activity in combination with antibiotics, reducing efflux activity and restoring intracellular antibiotic concentrations in MDR strains (Sharma et al., 2020; Dias et al., 2022). While much of the literature on EPIs involves plant-derived terpenoids, numerous bacteria also encode terpene BGCs (and microbially produced terpenoids) that exert similar activities. EPIs are measured using ethidium bromide accumulation assays, where increased intracellular fluorescence reflects impaired efflux (Zai et al., 2025). For bacterial EPIs, fluorescent dye accumulation assays with *E. coli* or *P. aeruginosa* efflux pump overexpressing strains show 40–100% inhibition of efflux at sub-MIC terpene concentrations (Dias et al., 2022; Tabcheh et al., 2025); concurrent application with antibiotics (e.g., ciprofloxacin) reduces MICs by 4–8-fold depending on strain and terpene (Ton et al., 2025; Higazy et al., 2024).

#### 4.2.4 Alkaloids and small redox-active metabolites

Certain non-peptidic alkaloids (Figures 2d–g) and redox-active compounds modulate resistance by targeting cellular respiration, membrane potential, or specific resistance enzymes (Bhattacharya et al., 2025; Duan et al., 2025). Although much work on plant alkaloids is outside bacterial secondary metabolites, bacterial alkaloids (and phenazine-like redox metabolites) from *Pseudomonas* or *Burkholderia* show potential in perturbing biofilms and enhancing antibiotic activity (Williams et al., 2025). For example, phenazine production is linked to inter-species competition and can disrupt electron transport chains, weaken tolerant cells, and sensitize them to oxidative stress. Phenazines combined with conventional antibiotics *in vitro* biofilm models reduce viability more effectively than either agent alone, often achieving synergy indices (e.g., fractional inhibitory concentration index) consistent with enhanced killing (Demisie et al., 2024).

### 4.3 Biosynthetic gene clusters: genomic drivers of metabolite innovation

Biosynthetic gene clusters (BGCs) encode the enzymatic machinery that drives the synthesis, variation, and innovation of secondary metabolites. NRPS and PKS clusters are wealthy sources of structural diversity: each module predicts a building block and tailoring domain, allowing in silico prediction of likely chemical backbones (McBride et al., 2023; Li et al., 2024; Sigrist et al., 2020).

Recent advances in genome mining and antiSMASH-style annotation have linked BGCs to previously uncharacterized metabolites with bioactivity, enabling prioritization for chemical isolation and functional testing. These clusters often contain regulatory genes that respond to environmental cues (e.g., quorum-sensing signals, nutrient status), explaining why many BGCs are “silent” under laboratory conditions and require elicitation or heterologous expression for metabolite production (Peng et al., 2021; Leal et al., 2025).

Understanding BGC architecture informs SAR (structure–activity relationship) studies (Ancajas et al., 2024): altering substrate specificity in adenylation (NRPS) or acyltransferase (PKS) domains can generate analogs with improved anti-resistance activity (e.g., enhanced efflux pump inhibition or altered membrane interaction). Genome-based cluster families (GCFs) that map to related chemical scaffolds serve as valuable leads in discovery pipelines linking genomic potential to functional activity (Navarro-Muñoz et al., 2020; Sahana et al., 2025).

### 4.4 Ecological rationale for metabolite production: mechanistic implications for anti-resistance activity

While secondary metabolites originate from ecological selection, their relevance to antimicrobial resistance lies in the mechanistic features encoded by these evolutionary pressures. Metabolites shaped by competition, signalling, and community dynamics frequently engage bacterial processes that are directly implicated in resistance and tolerance, providing a mechanistic bridge between ecology and therapeutic utility.

#### 4.4.1 Chemical defense as a source of non-canonical resistance targets

Metabolites evolved for chemical defense often interfere with fundamental cellular functions required for survival under competitive stress. Importantly, these functions frequently overlap with pathways co-opted by resistant bacteria, including membrane integrity, energy homeostasis, efflux activity, and translational control. Ecologically derived antimicrobial metabolites therefore tend to act through non-canonical binding sites or physicochemical mechanisms, reducing cross-resistance with conventional antibiotics. This principle is exemplified by soil-derived lasso peptides such as lariocidin, which inhibit protein synthesis through ribosomal interactions distinct from those targeted by classical translation inhibitors. Structural and biochemical analyses demonstrate that such metabolites bind unique ribosomal pockets, preserving activity against pathogens resistant to aminoglycosides or macrolides (Mucha et al., 2025). More broadly, metabolites evolved for defense frequently exhibit multifactorial modes of action, imposing higher barriers to resistance emergence than single-target antibiotics (Vareschi et al., 2025).

#### 4.4.2 Signalling interference and suppression of resistance-associated phenotypes

Many secondary metabolites function as analogs or antagonists of quorum-sensing signals, and their mechanistic relevance lies in their ability to modulate regulatory networks that control resistance-associated traits. Disruption of quorum-sensing pathways downregulates virulence factor production, biofilm maturation, and coordinated stress responses, indirectly increasing susceptibility to antimicrobial agents. Experimental studies show that quorum-sensing antagonists reduce expression of efflux pumps and biofilm-associated tolerance mechanisms, leading to enhanced antibiotic penetration and activity (Chu and Yang, 2024). Synthetic and semi-synthetic derivatives of naturally occurring signalling metabolites have been developed to competitively inhibit quorum-sensing receptors, resulting in substantial reductions in biofilm biomass and antibiotic tolerance in multidrug-resistant *Pseudomonas aeruginosa* models (Wright and Ramachandra, 2022; Hetta et al., 2024; Bernabè et al., 2025). These findings highlight signalling interference as a mechanistically indirect but clinically relevant anti-resistance strategy.

#### 4.4.3 Competition, resource control, and destabilization of resistant communities

Secondary metabolites involved in competitive resource acquisition exert mechanistic effects that destabilize resistant populations at the community level. Siderophores, for example, sequester iron and deprive competing organisms of a cofactor essential for metabolic enzymes, oxidative stress defense, and certain resistance determinants. Iron limitation has been shown to impair activity of iron-dependent enzymes and weaken biofilm-associated tolerance, sensitizing bacteria to antimicrobial stress. In parallel, metabolites that influence extracellular matrix production or degradation can modulate cooperative behaviors that shield resistant cells from antibiotics. While cooperative production of extracellular polysaccharides may enhance collective tolerance, these structures also create vulnerabilities that can be exploited by metabolic adjuvants or matrix-disrupting agents. Mechanistically, such interventions increase antibiotic diffusion, reduce local resistance niches, and collapse protective community architectures.

**Figure 3:**
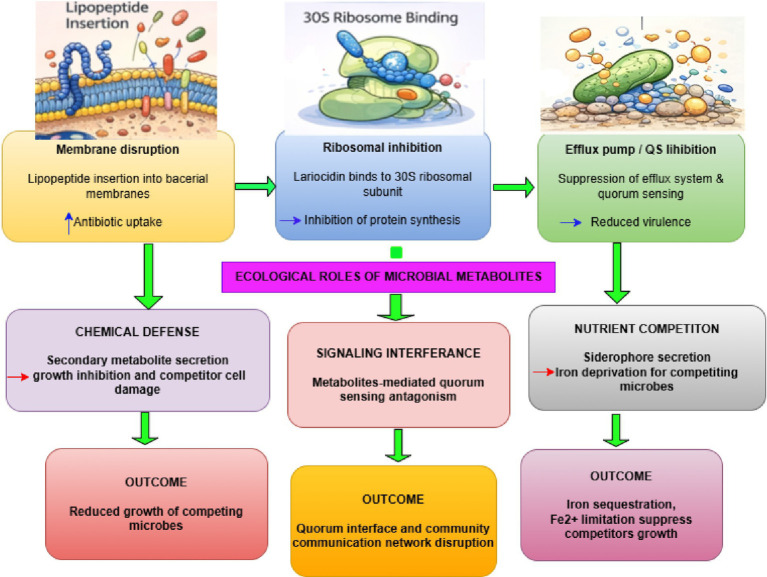
Mechanisms of antibacterial secondary metabolites.

Together, these mechanisms demonstrate that metabolites evolved for competition and cooperation can be repurposed to disrupt resistance-supporting microenvironments, extending their relevance beyond individual cells to population-level resistance dynamics (Figure 3; Tables 2, 3).

*Membrane disruption*: Amphiphilic bacterial secondary metabolites, such as cyclic lipopeptides and polyketide-derived surfactants, insert into bacterial membranes, increasing permeability and dissipating the proton motive force. This compromises barrier function, enhances intracellular antibiotic accumulation, and weakens membrane-associated resistance mechanisms, thereby potentiating bactericidal activity.
*Ribosomal inhibition*: Ribosomally synthesized and post-translationally modified peptides (RiPPs), exemplified by lasso peptides such as lariocidin, bind non-classical sites on the bacterial ribosome. By interfering with tRNA accommodation and translocation at the 30S subunit, these metabolites arrest protein synthesis and retain activity against strains harboring conventional ribosomal resistance mutations.
*Efflux pump inhibition*: Certain bacterial secondary metabolites act as efflux pump inhibitors by blocking transporter binding pockets or disrupting the energy gradients that drive drug extrusion. Inhibition of RND, MFS, or ABC transporters increases intracellular antibiotic retention, resulting in reduced MICs and restoration of susceptibility in multidrug-resistant bacteria.
*Quorum sensing inhibition*: Secondary metabolites that mimic or antagonize quorum-sensing signals disrupt bacterial communication networks. By preventing activation of virulence-and resistance-associated regulons, these compounds suppress coordinated behaviors such as toxin production and biofilm maturation, indirectly enhancing antibiotic effectiveness.
*Biofilm disruption*: Biofilm-disrupting metabolites degrade extracellular polymeric substances or inhibit matrix assembly, leading to biofilm destabilization and dispersal. This reduces diffusion barriers, increases antibiotic penetration, and resensitizes biofilm-associated bacterial populations that are otherwise tolerant to antimicrobial treatment.
*Chemical defense*: Bacterial secondary metabolites function as chemical weapons in competitive microbial environments, suppressing or eliminating neighboring organisms. These inhibitory compounds confer ecological advantage by limiting resource competition and shaping community composition, and their evolved bioactivities can be repurposed therapeutically to target resistant pathogens.
*Signaling*: Many secondary metabolites act as signaling molecules that regulate population density, gene expression, and collective behaviors such as virulence and biofilm formation. By mediating quorum sensing and interspecies communication, these metabolites coordinate adaptive responses to environmental cues and stressors, influencing microbial fitness and persistence.
*Nutrient competition*: Metabolites such as siderophores mediate competition for essential nutrients, particularly iron, by sequestering scarce resources from the environment. This nutrient deprivation strategy restricts competitor growth and survival, and in pathogenic contexts can weaken bacterial defenses and enhance susceptibility to antimicrobial intervention.

**Table 2:** Representative bacterial secondary metabolites with experimentally validated anti-resistance activity.

Metabolite/class	Producer/source	Mechanism of anti-resistance action	Experimental outcomes (MDR context)	Reference(s)
Lariocidin (lasso peptide, RiPP)	*Paenibacillus* sp. M2	Ribosomal inhibition (unique small-subunit binding); not affected by common resistance mechanisms	Broad-spectrum growth inhibition, including *Acinetobacter baumannii*, *Klebsiella pneumoniae,* and *E. coli* resistant strains; potent *in vivo* efficacy in the mouse *A. baumannii* infection model	Jangra et al. (2025)
Corramycin (NRPS-PKS hybrid)	*Corallococcus coralloides* (myxobacteria)	Novel antibacterial scaffold with activity against Gram-negatives; minimal cross-resistance observed	Anti-*E. coli* activity *in vivo* (mouse models of systemic infection) with reduced CFUs and survival benefit; low observed resistance frequency	Couturier et al. (2022), Adam et al. (2024)
Pyoluteorin (NRPS/PKS)	*Pseudomonas fluorescens*	Membrane and multisite antimicrobial actions (historical plant pathogen context)	Though originally characterized for plant pathogen suppression, pyoluteorin-class metabolites represent NRPS/PKS engagement and structural classes used in subsequent optimization strategies against resistant bacteria.	Pellicciaro et al. (2022), Rose et al. (2021)
Loeseneriella africana terpenoid/β-sitosterol derivatives (plant-associated)	Endophytic/associated bacteria (constituents from plant sources)	Efflux pump inhibition, biofilm disruption, resistance modulation	Isolated terpenoids significantly inhibited efflux activity and biofilms in *E. coli* and *P. aeruginosa* (efflux inhibition ≥87–110%), and enhanced amoxicillin activity with modulation factors ≥10–32	Anokwah et al. (2024)

Lariocidin is among the first validated RiPPs with direct ribosome targeting and has shown *in vivo* efficacy against MDR Gram-negative bacteria.

Corramycin is a promising NRPS-PKS hybrid natural product with confirmed bactericidal effects against Gram-negative pathogens in animal models and minimal. Cross-resistance, although its precise molecular target remains under investigation.

Entries like pyoluteorin exemplify metabolite classes that display structural and ecological diversity; data on resistance modulation may be found in derivative studies.

**Table 3:** Biosynthetic gene cluster (BGC) features and their functional implications for anti-resistance metabolite innovation.

BGC feature	Biosynthetic implication	Predictive value for anti-resistance activity	Key notes/references
NRPS modules (adenylation/condensation/thiolation)	Enable assembly of cyclic lipopeptides and complex peptides with amphiphilic properties	Amphiphilic peptides (CLPs) can disrupt membranes and potentiate antibiotic penetration; they may reduce tolerance phenotypes	NRPS diversity underlies many membrane-active antimicrobial scaffolds; implied mechanistic relevance (Allemailem, 2021)
PKS domains (ketosynthase, acyltransferase)	Build polyketide backbones with structural complexity	Polyketide scaffolds can interact with cell envelopes and metabolic targets distinct from classical antibiotic sites	PKS gene presence correlates with novel structural scaffolds in Myxobacterial metabolites (Couturier et al., 2022)
Hybrid NRPS-PKS clusters	Combine peptide and polyketide elements	Provide chemical space for unique antibacterial agents (e.g., Corramycin) with activity against MDR Gram-negatives.	Corramycin BGCs yield structurally novel molecules with *in vivo* efficacy (Siebert and Kazmaier, 2024)
RiPP biosynthetic clusters (lasso peptide)	Encode precursor peptides and tailoring enzymes, conferring high structural stability	RiPPs like lariocidin bind novel targets (ribosome) and evade common resistance determinants.	Lariocidin BGCs encode maturation enzymes and unusual topology associated with the mechanism (Jangra et al., 2025)
Transporter and self-resistance genes within BGC loci	Indicate biological roles and necessary immunity functions	Presence of resistance/self-protection genes suggests potent bioactivity and requirement for export/ sequestration	Xia et al., 2020; Crits-Christoph et al., 2021
Silent/cryptic regulatory elements	BGCs under tight regulation often require elicitation	Silent clusters represent untapped reservoirs of metabolites with potential anti-resistance activity upon activation	Large genomic surveys reveal many silent BGCs awaiting functional characterization. Deshmukh et al. (2023), Covington et al. (2021), Pillay et al. (2022)

Legend and Usage: This table links BGC architectural features to their functional roles in producing metabolites of interest for anti-resistance strategies (e.g., membrane disruption, ribosome targeting, efflux modulation).

Features like hybrid NRPS-PKS architectures often correlate with structurally novel compounds capable of circumventing existing resistance mechanisms.

RiPP clusters, exemplified by lariocidin BGCs, demonstrate how post-translational modifications can generate molecules with mechanisms distinct from classical. Antibiotics diminish the propensity for rapid resistance emergence.

## 5 Molecular mechanisms by which metabolites function as anti-resistance modalities

Bacterial secondary metabolites counter antimicrobial resistance not merely by exerting bactericidal pressure, but by directly targeting resistance determinants, destabilizing tolerance phenotypes, modulating host–pathogen interactions, and restoring antibiotic susceptibility through synergistic mechanisms. Unlike conventional antibiotics, many metabolites evolved under ecological selection pressures (ecological metabolites or specialized metabolites) favoring interference with microbial competitiveness, signaling, and survival, making them particularly well-suited to disrupt resistance architectures that are poorly addressed by classical drugs (Mahon et al., 2025; Sam et al., 2024).

### 5.1 Direct interference with canonical resistance mechanisms

Unlike the ecological and evolutionary rationales outlined in Sections 3.3 and 4.3, this section focuses exclusively on direct molecular and biochemical interactions by which secondary metabolites disable established antimicrobial resistance mechanisms. These activities do not rely on indirect ecological effects or community-level modulation but instead involve measurable inhibition of resistance-conferring proteins or pathways, resulting in restored antibiotic susceptibility.

#### 5.1.1 Efflux pump inhibition

Active efflux represents a dominant mechanism of multidrug resistance, particularly in Gram-negative pathogens, where resistance-nodulation-division (RND), major facilitator superfamily (MFS), and ATP-binding cassette (ABC) transporters reduce intracellular antibiotic concentrations below therapeutic thresholds (Davin-Regli et al., 2021). Functional inhibition of these transporters directly enhances intracellular drug accumulation and constitutes a validated anti-resistance strategy.

Multiple studies demonstrate that secondary metabolites can suppress efflux activity as quantified by ethidium bromide or Nile red accumulation assays, where increased intracellular fluorescence reflects transporter inhibition (Pal et al., 2020; Seukep et al., 2020). Natural compounds from both microbial and plant sources have shown activity across multiple transporter families, rather than acting as narrow-spectrum pump blockers. For example, ethyl acetate fractions enriched in triterpenoids from *Loeseneriella africana* reduced efflux activity by >80% at sub-inhibitory concentrations in *Escherichia coli* and *Pseudomonas aeruginosa*, with concomitant potentiation of fluoroquinolones and *β*-lactams (Anokwah et al., 2024).

Mechanistically, efflux inhibition may occur through direct binding to transporter cavities or disruption of energy coupling, particularly proton motive force–dependent transport. Structural and biochemical analyses implicate RND systems such as AcrAB–TolC and MexAB–OprM, as well as MFS (EmrAB) and ABC (MacAB–TolC) transporters, all of which span the Gram-negative cell envelope and extrude chemically diverse antibiotics (Rouvier et al., 2025). Docking and cryo-EM–guided studies suggest that phenolic and terpenoid metabolites preferentially occupy access or deep binding pockets within RND transporters, competitively interfering with substrate extrusion (Huang et al., 2022; Pos, 2024). Functionally, efflux inhibition frequently yields 4–16-fold reductions in antibiotic MICs, particularly for fluoroquinolones and tetracyclines, without imposing strong bactericidal pressure (Zhang L. et al., 2024).

#### 5.1.2 Suppression of *β*-lactamase activity

β-Lactamase-mediated antibiotic inactivation remains a major driver of resistance, particularly among Enterobacteriaceae and non-fermenting Gram-negative pathogens. Extended-spectrum β-lactamases (ESBLs) and metallo-β-lactamases (MBLs), including NDM-1 and VIM-2, hydrolyze β-lactams and carbapenems, severely limiting therapeutic options (Boyd et al., 2020). Direct inhibition of these enzymes represents a high-value anti-resistance intervention.

Several secondary metabolites have demonstrated direct β-lactamase inhibitory activity in biochemical assays. Phenolic polyketides and alkaloid-like scaffolds have been shown to bind near catalytic serine residues in class A enzymes or to interfere with zinc coordination in class B MBLs, resulting in reduced hydrolytic activity (Huang and Zhou, 2025; Sargianou et al., 2025). Enzyme kinetics assays reveal substantial decreases in catalytic efficiency, while microbiological combination studies confirm restoration of β-lactam susceptibility in ESBL-producing *E. coli* and *Klebsiella pneumoniae*.

#### 5.1.3 Inhibition of Cell wall remodelling and peptidoglycan turnover

Adaptive remodelling of the bacterial cell wall—including altered peptidoglycan cross-linking, modified autolysin activity, and changes in penicillin-binding proteins (PBPs)—contributes to resistance against β-lactams and glycopeptides. Beyond inhibition of canonical biosynthetic enzymes, emerging evidence indicates that interference with peptidoglycan turnover and recycling can sensitize resistant strains to existing antibiotics. Certain ribosomally synthesized and post-translationally modified peptides (RiPPs) and non-ribosomal peptides interfere with lipid II availability or disrupt MurA/MurB-associated pathways, indirectly weakening cell wall integrity (Cao et al., 2021). By perturbing peptidoglycan recycling or autolysin regulation, these metabolites amplify the activity of β-lactams and glycopeptides even in strains harboring altered PBPs. Related mechanistic effects have been observed with natural depsipeptides and glycopeptide analogs that alter envelope dynamics, contributing to synergy with cell wall–targeting drugs (Kumar, 2024).

While systematic evaluation of this mechanism in clinical MDR isolates remains limited, existing biochemical and microbiological evidence supports cell wall turnover as a tractable resistance-modifying target for secondary metabolites. Focused validation in resistant backgrounds is a priority for future work.

#### 5.1.4 Targeting ribosomal protection proteins and resistance-associated mutations

Ribosomal protection proteins and target-site mutations compromise the efficacy of translation-targeting antibiotics such as macrolides and aminoglycosides. Recent discoveries demonstrate that certain secondary metabolites can bypass these resistance mechanisms by engaging alternative ribosomal binding sites.

Lasso peptides such as lariocidin bind the 30S ribosomal subunit and inhibit translation by blocking tRNA accommodation and translocation. Structural, biochemical, and microbiological studies show that lariocidin retains activity against priority pathogens including *Acinetobacter baumannii*, *Klebsiella pneumoniae*, *Staphylococcus aureus*, and *E. coli*, despite resistance to conventional ribosome-targeting antibiotics (Mucha et al., 2025; Jangra et al., 2025). Its binding mode is distinct from that of macrolides or aminoglycosides, allowing it to circumvent common resistance mutations and ribosomal protection factors.

These findings illustrate how secondary metabolites can exploit previously underutilized ribosomal vulnerabilities, offering a direct and mechanistically validated route to overcoming translation-associated resistance.

### 5.2 Disruption of biofilms and quorum sensing

#### 5.2.1 Metabolites that degrade biofilm EPS or inhibit matrix assembly

Biofilms confer profound antibiotic tolerance due to extracellular polymeric substance (EPS) barriers. Natural compounds such as phytochemicals (e.g., flavonoids, terpenoids) disrupt biofilm structural integrity and inhibit formation. Multiple *in vitro* studies report significant reductions in biofilm biomass upon treatment with these metabolites, accompanied by enhanced antibiotic penetration (Fydrych et al., 2025). Phenazine derivatives, cyclic lipopeptides, and glycoside-like metabolites reduce biofilm biomass by 40–80% in vitro, often restoring antibiotic susceptibility without directly killing planktonic cells (Sousa et al., 2024; Huang et al., 2024). Assays directly measuring biofilm disruption use crystal violet staining, confocal microscopy, and viability counts, showing weakened EPS matrix and compromised community resilience.

#### 5.2.2 Quorum-sensing antagonists and signal mimicry

Quorum sensing (QS) regulates biofilm formation, virulence, and resistance gene expression. A 2025 primary research review catalogues natural product–based QS inhibitors (QSIs) derived from plants, microbes, and marine organisms that act by inhibiting signal synthesis, blocking receptor binding, or degrading signaling molecules, leading to reduced virulence and enhanced antibiotic efficacy (Alum et al., 2025). Small cyclic peptides, diketopiperazines, and halogenated metabolites have been shown to suppress QS-regulated phenotypes in *P. aeruginosa* and *Staphylococcus aureus*, leading to reduced toxin production and enhanced antibiotic susceptibility (Arendse et al., 2022; Alam et al., 2020). Examples from this literature include flavonoids, alkaloids, and diketopiperazines with validated QS interference activity in *P. aeruginosa* and other pathogens, demonstrating reduced QS-regulated phenotypes and potentiation of antibiotics in vitro.

#### 5.2.3 Re-sensitization of biofilm-associated MDR pathogens

QS disruption and EPS degradation jointly convert biofilm-embedded tolerant populations into antibiotic-responsive states (Sajeevan et al., 2025). Combination studies using natural QSIs and conventional antibiotics have shown synergistic reductions in MBIC (minimum biofilm inhibitory concentrations) and improved eradication of MDR biofilms (Hawas et al., 2022; Wang J. et al., 2024), although more primary experimental citations are needed for specific microbial metabolites.

### 5.3 Immunomodulatory metabolites that restore host control

#### 5.3.1 Metabolites enhancing phagocytosis and bacterial clearance

Some microbial metabolites modulate host immune responses, enhancing macrophage phagocytosis and neutrophil activity (O’Callaghan et al., 2021; Dang et al., 2023). Although mechanistic data specific to secondary metabolites contexts are limited, evidence supports immunomodulation as a viable anti-resistance adjunct strategy.

#### 5.3.2 Modulating inflammatory cascades to counteract persistence phenotypes

Excessive or dysregulated inflammation facilitates persistence phenotypes. Natural small molecules that modulate inflammatory signaling (e.g., NF-κB, MAPK pathways) can reduce bacterial survival niches (Jenab et al., 2020). While detailed primary studies directly linking specific metabolites to improved clearance in resistant infections remain emerging, this immunomodulatory mechanism is increasingly recognized in infection biology research.

#### 5.3.3 Anti-virulence metabolites neutralizing toxin production

Anti-virulence strategies aim to neutralize pathogenic factors without imposing strong selective pressure. Certain secondary metabolites downregulate toxin gene expression and secretion systems, weakening pathogens and enhancing antibiotic susceptibility (Perry et al., 2022). Recent studies support natural virulence modulation as a resistance-bypassing approach (Křížkovská et al., 2023; McCarthy et al., 2021), although specific compound–pathogen pairings require further empirical study.

### 5.4 Synergy with existing antibiotics

#### 5.4.1 Mechanistic basis for metabolic–antibiotic synergy

Synergy arises when natural metabolites enhance antibiotic effectiveness through combined mechanisms such as efflux inhibition, membrane permeabilization, or resistance enzyme suppression. Primary research pairs specific natural compounds with antibiotics to quantify synergy via checkerboard assays and fractional inhibitory concentration (FIC) indices, supporting the combinatorial use of metabolites and drugs (Malczak and Gajda, 2023).

5.4.2 Case Studies: Aminoglycoside Potentiation and *β*-Lactam Re-sensitization.

Natural compounds that disrupt efflux and membrane barriers have been shown to enhance aminoglycoside uptake and β-lactam susceptibility in resistant models (Kumar et al., 2025), although the most detailed examples remain plant-derived flavonoids and terpenoids rather than pure bacterial metabolites. Continued research is needed to identify specific bacterial metabolite–antibiotic pairs with quantified synergy in MDR pathogens.

#### 5.4.2 Metabolite-driven disruption of pathogen metabolic homeostasis

Secondary metabolites that perturb core metabolic pathways (e.g., redox balance, energy production) create physiological stress that sensitizes bacteria to antibiotics (Perry et al., 2022; Arce-Rodríguez et al., 2022). While mechanistic proposals are grounded in bacterial physiology studies, direct experimentally validated examples for discrete bacterial metabolites remain a growing area of research.

## 6 Evolutionary dynamics: how secondary metabolites shape resistance landscapes

Understanding how bacterial secondary metabolites influence the evolution and maintenance of resistance determinants is essential to rationally deploy these molecules as anti-resistance modalities. Evolutionary dynamics occur across scales—from molecular target mutational pathways, through physiological trade-offs at the cellular level, to ecological selection in complex communities—and each scale informs design principles for evolution-resilient therapeutics. Below, we synthesize current evidence on co-evolutionary history, propensity for resistance emergence, fitness trade-offs, and ecological lessons applicable to therapeutic design.

### 6.1 Co-evolution of natural antibiotics and resistance determinants

Antibiotics and resistance determinants share a deep evolutionary history: many resistance genes predate anthropogenic antibiotic use and evolved under natural selection exerted by microbial secondary metabolites in environmental microbiomes (Selvarajan et al., 2022; Larsson and Flach, 2022). Genomic and metagenomic surveys demonstrate that environmental reservoirs (soil, aquatic, and sewage settings) harbor a rich “resistome” of enzymes, efflux pumps, and modification systems that can be mobilized into pathogens via horizontal gene transfer (HGT). These findings underscore that environmental exposure to natural products has historically selected for cognate resistance mechanisms long before clinical antibiotic deployment (Muteeb et al., 2023; Ma et al., 2024).

Recent syntheses argue that the co-existence of biosynthetic gene clusters (BGCs) and resistance loci within the same or neighboring genomes is common (Kwon et al., 2021): producers often carry self-protection genes that neutralize or export their own metabolites, creating a local selective regime that maintains resistance determinants in environmental gene pools (Yan et al., 2020; Knowles et al., 2022). The genomic colocation of BGCs and resistance/self-protection elements provides a molecular record of co-evolution and a rationale for why some resistance mechanisms are pervasive across taxa (Muteeb et al., 2023).

*Implication for therapeutics:* knowledge of environmental resistome and historical co-evolution should guide compound selection and prioritization. Molecules whose producing organisms lack obvious self-protection genes against the mechanism of interest, or whose BGC families are rare in environmental metagenomes, are—by this logic—less likely to encounter pre-existing resistance determinants in clinical populations.

### 6.2 Why many metabolites avoid rapid resistance development

Several ecological and mechanistic properties of natural metabolites reduce the probability that simple, high-frequency mutations will generate cross-resistance:

#### 6.2.1 Multimodal or non-single-target actions

Many secondary metabolites act on membranes, disrupt energy generation, or perturb multiple cellular processes simultaneously, making single-site mutations less likely to confer high-level resistance (Gorlenko et al., 2020; Suganya et al., 2022). Compounds with broad biophysical modes (e.g., amphiphilic lipopeptides) typically require complex, costly compensatory changes for meaningful resistance. This principle is supported by experimental evolution and theoretical models showing slower resistance emergence to multi-target agents compared with single-target antibiotics (Pinheiro, 2024; Chowdhury and Findlay, 2023).

#### 6.2.2 Lower selective pressure when used as adjuvants/anti-virulence agents

Anti-virulence strategies and adjuvant use reduce direct lethality while impairing pathogenicity or resistance mechanisms (e.g., biofilm dispersal, efflux inhibition) (Daher et al., 2025). Such approaches are predicted to impose weaker selection for resistance than bactericidal therapy because they do not drastically reduce population size in a way that favors resistant mutants—a point repeatedly argued in the anti-virulence literature (Dehbanipour and Ghalavand, 2022).

#### 6.2.3 Ecologically co-occurring combinatorial chemistry

In natural environments, microbes are exposed to complex mixtures of metabolites rather than single compounds; therefore, resistance must often be broad to be advantageous (Elshobary et al., 2025; Vaou et al., 2021). This “cocktail” context selects for generalist resistance strategies (e.g., robust stress responses) rather than single-point mutations, which can be more costly and less likely to spread. Analyses of environmental metagenomes and metabolomes support widespread combinatorial exposures that constrain simple resistance evolution (Ma et al., 2024).

*Intrinsic fitness costs of resistance–*Mutations conferring resistance to certain metabolite classes often reduce growth rate or competitiveness in drug-free environments; when costs are high, selection will purge resistance alleles in the absence of continuous exposure, slowing their dissemination (Hemez et al., 2022). Experimental work demonstrates substantial fitness costs for some resistance mechanisms, and modeling predicts slower evolution under these constraints (Pinheiro, 2024; Chowdhury and Findlay, 2023). These factors together explain why some natural metabolites appear to show low rates of resistance emergence in experimental studies. However, it is important to note that exceptions exist and that compensatory evolution, HGT, and co-selection by other stressors may still enable resistance spread.

### 6.3 Adaptive trade-offs and fitness costs in metabolite-resistant mutants

Resistance mutations frequently incur trade-offs–reductions in growth rate, metabolic efficiency, or stress tolerance—that shape their evolutionary trajectories. Empirical studies quantify these costs and show how compensatory mutations can modulate them.

- *Measured fitness costs:* Experimental evolution and competition assays reveal that resistance to particular drugs (and to some natural products) lowers relative fitness in antibiotic-free media; for example, resistance mutations in ribosomal genes or membrane-associated loci can impair translational efficiency or membrane integrity, respectively. These fitness costs directly influence whether resistance will persist once selection is relaxed (Dhital et al., 2025).
- *Compensatory evolution:* Over time, secondary mutations can ameliorate fitness costs while retaining resistance, enabling persistence and spread. Genomic surveillance indicates that compensatory changes are commonly observed in clinical lineages, reducing the long-term advantage of relying solely on fitness costs to limit resistance (Hossain and Chowdhury, 2024).
- *Ecological context dependence*: Fitness effects are environment-dependent: a mutation that is costly in minimal laboratory media may be neutral or advantageous in complex host or environmental niches (e.g., within biofilms or nutrient-rich host tissues), facilitating persistence of resistant genotypes (Manktelow et al., 2020). This context dependence requires that evolutionary assessments of candidate metabolites include realistic ecological models (biofilms, host environments), not just planktonic assays (Soley et al., 2023).
- *Implication for drug design:* therapeutic strategies should aim to couple high fitness costs to resistance with limited opportunities for compensatory evolution, for example, by targeting essential, pleiotropic processes or combining modalities that jointly constrain compensatory pathways.

### 6.4 Ecological insights for designing evolution-resilient anti-resistance therapeutics

Ecology supplies concrete design principles for minimizing resistance emergence and spread when employing secondary metabolites clinically:

- *Prioritize multimodal and context-specific mechanisms:* Molecules that act via membrane perturbation, energy collapse, or simultaneous attack on multiple pathways are intrinsically harder for bacteria to evade via single mutations (Wu et al., 2024). The ecological prevalence of such multitarget compounds suggests they are evolutionarily robust, an observation supported by comparative resistance evolution studies (Matei and Visan, 2025).
- *Use metabolites as adjuvants or anti-virulence agents rather than sole bactericidal drugs:* When used to potentiate existing antibiotics (e.g., EPIs, *β*-lactamase suppressors, biofilm dispersers), metabolites can reduce required antibiotic doses and alter selection regimes, potentially lowering the rate of resistance evolution. Modeling and empirical data on anti-virulence strategies support reduced selective pressure under this paradigm, though exceptions exist and continued surveillance is essential (Dehbanipour and Ghalavand, 2022; Murray et al., 2024).
- *Exploit ecological incompatibilities and collateral sensitivity networks:* Evolutionary trade-offs sometimes produce collateral sensitivity, where resistance to one compound increases sensitivity to another. Mapping collateral sensitivity networks experimentally enables rational pairing of metabolites and antibiotics to steer evolution away from resistant states. Recent theoretical and empirical work demonstrates the promise of evolution-informed combination therapy design (Pinheiro, 2024).
- *Target environments with limited opportunities for HGT and co-selection*: Deploying evolution-resilient therapeutics in contexts where horizontal transfer is constrained (e.g., acute care settings with strict infection control) can reduce the risk that resistance genes disseminate (Elbehiry et al., 2025; Reynolds, 2025; El-Sayed et al., 2026). Conversely, environmental release (e.g., agricultural application) demands extreme caution because co-selection via metals, biocides, or other contaminants can rapidly fix resistance determinants—a point highlighted by recent co-selection reviews (Gillieatt and Coleman, 2024).
- *Include realistic ecological models in preclinical pipelines:* Candidate metabolites should be tested in biofilm models, polymicrobial communities, and *in vivo* infection models that better recapitulate selection dynamics, compensatory mutation opportunities, and HGT likelihood—not only in planktonic monocultures. Studies of environmental resistome and wastewater microbiomes illustrate that community context substantially alters selection trajectories (Ma et al., 2024).

## 7 Discovery, mining, and optimization of anti-resistance metabolites

The discovery of bacterial secondary metabolites with anti-resistance activity has shifted from serendipitous screening toward integrated, data-driven discovery pipelines. Advances in genome mining, multi-omics integration, synthetic biology, and artificial intelligence now enable systematic identification, mechanistic annotation, and optimization of metabolites that disrupt resistance phenotypes rather than simply inhibit growth. This section outlines the contemporary methodological landscape supporting the discovery and development of evolution-resilient anti-resistance metabolites.

### 7.1 Genome mining and multi-omics tools

#### 7.1.1 BGC mining with antiSMASH, PRISM, and deep-learning models

Genome mining has become a cornerstone of natural product discovery following the recognition that most bacterial secondary metabolites are encoded by biosynthetic gene clusters (BGCs). Tools such as antiSMASH and PRISM enable automated detection, annotation, and classification of BGCs from bacterial genomes and metagenomes. antiSMASH identifies conserved biosynthetic domains (e.g., NRPS, PKS, RiPP enzymes), predicts cluster boundaries, and links BGCs to known chemical families through comparison with curated databases such as MIBiG (Blin et al., 2023). Continuous updates have expanded coverage to include hybrid clusters, tailoring enzymes, and regulatory elements, improving prioritization of clusters likely to encode novel chemistry (Blin et al., 2025). PRISM complements antiSMASH by reconstructing putative chemical structures from BGC architecture, enabling in silico prediction of scaffold novelty and functional moieties that may be relevant to resistance modulation (Skinnider et al., 2020). Together, these tools allow early triaging of BGCs based on predicted structural complexity, biosynthetic logic, and divergence from known antibiotic classes. More recently, deep-learning–assisted genome mining has been introduced to overcome limitations of rule-based annotation (Chen Z. et al., 2024). Neural-network models trained on curated BGC datasets can identify atypical or cryptic clusters lacking canonical motifs and predict bioactivity-associated features beyond chemical class alone (Zhu et al., 2025). These approaches are particularly valuable for identifying metabolites with non-classical mechanisms, such as efflux modulation or biofilm interference, which are not readily inferred from scaffold class alone.

#### 7.1.2 Metabolomics (LC–MS and MS/MS networking) for metabolite discovery

Genome mining alone cannot confirm metabolite production or function; therefore, mass spectrometry–based metabolomics is essential for connecting BGCs to chemical entities. High-resolution LC–MS coupled with tandem MS (MS/MS) enables detection of secondary metabolites across diverse growth conditions (Das et al., 2023). The development of Global Natural Products Social Molecular Networking (GNPS) has transformed metabolomics by organizing MS/MS spectra into molecular networks based on structural similarity, facilitating dereplication and discovery of novel analogs (Nothias et al., 2020; Qin et al., 2023). Molecular networking is particularly powerful when combined with paired-omics strategies, where predicted BGC products are correlated with MS features. This integration accelerates the identification of metabolite families, reveals biosynthetic diversification, and highlights minor congeners that may possess superior anti-resistance activity. Importantly, metabolomics-based discovery captures condition-dependent metabolite production, including metabolites induced only under stress, co-culture, or sub-inhibitory antibiotic exposure—conditions relevant to resistance ecology.

#### 7.1.3 Transcriptomics and proteomics for mechanism elucidation

While metabolomics identifies chemical entities, transcriptomics and proteomics are essential for elucidating mechanisms of action. RNA sequencing enables assessment of global transcriptional responses to metabolite exposure, revealing whether resistance determinants (e.g., efflux pumps, *β*-lactamases, biofilm genes) are directly inhibited, downregulated, or bypassed (Kaynar et al., 2021). Proteomic profiling further clarifies effects on protein abundance, post-translational modification, and stress-response pathways (Zhai et al., 2022). Integrated transcriptomic–proteomic analyses have been used to distinguish bactericidal activity from resistance modulation (Aita et al., 2025), for example, by demonstrating suppression of efflux pump expression or quorum-sensing regulons without induction of classical cell death pathways (Lin et al., 2023; Hetta et al., 2024). Such mechanistic resolution is critical for validating metabolites as anti-resistance adjuvants rather than conventional antibiotics.

### 7.2 Synthetic biology and bioengineering approaches

#### 7.2.1 Heterologous expression and pathway refactoring

A major bottleneck in natural product research is that many BGCs are silent or poorly expressed in their native hosts. Heterologous expression in genetically tractable hosts (e.g., *Streptomyces*, *Escherichia coli*, *Bacillus* spp.) enables controlled production, scalable yields, and systematic pathway manipulation (Lasch et al., 2025; Vojnovic et al., 2024; Lee et al., 2024). Advances in promoter engineering, codon optimization, and regulatory rewiring allow pathway refactoring, decoupling metabolite production from native regulatory constraints (Pu and Zhang, 2025). For anti-resistance research, heterologous systems facilitate rapid testing of metabolite libraries against resistant pathogens and enable structure–function studies that would be infeasible in slow-growing or genetically intractable producers.

#### 7.2.2 Engineering designer metabolites with improved potency

Synthetic biology enables rational modification of biosynthetic pathways to generate “designer” metabolites with enhanced anti-resistance activity (Chigozie et al., 2025a). Domain swapping in NRPS or PKS modules, alteration of tailoring enzymes, and precursor feeding strategies allow systematic variation of chemical features such as hydrophobicity, charge, or steric bulk—properties that influence membrane interaction, efflux susceptibility, and target binding (Fortinez et al., 2022; Iacovelli et al., 2021). Such combinatorial biosynthesis has yielded analogs with improved stability, reduced toxicity, and enhanced synergy with antibiotics in multiple systems (Yook et al., 2025). Importantly, optimization efforts increasingly prioritize resistance-modifying properties (e.g., efflux inhibition, biofilm disruption) rather than maximal bactericidal potency.

#### 7.2.3 CRISPR-guided manipulation of BGCs

CRISPR–Cas technologies have revolutionized the precise manipulation of BGCs, enabling targeted activation, deletion, or modification of biosynthetic genes (Krysenko, 2025). CRISPR interference (CRISPRi) allows tunable repression of pathway regulators to derepress silent clusters, while CRISPR–Cas9 editing facilitates domain-level engineering and removal of competing pathways (Sagharyan et al., 2025; Zhang et al., 2021). These tools accelerate functional validation of BGCs, enable rapid generation of metabolite variants, and reduce reliance on random mutagenesis. In anti-resistance discovery, CRISPR-guided approaches are particularly valuable for linking specific structural features to resistance-modulating activity.

### 7.3 AI-assisted metabolite discovery

#### 7.3.1 Predictive modeling of metabolite–target interactions

The rapid advancement of artificial intelligence (AI)—a computational discipline that enables machines to perform tasks traditionally reliant on human cognition—has ushered bioprospecting into a new phase of innovation. By leveraging machine learning, deep learning, and related data-driven approaches, AI offers transformative capabilities for the systematic discovery and prioritization of bioactive natural products (Chigozie et al., 2025b). Artificial intelligence is increasingly applied to predict metabolite–target interactions, particularly for non-classical targets such as efflux pumps, ribosomal sub-sites, and quorum-sensing receptors. Structure-based docking combined with machine-learning scoring functions enables prioritization of metabolites likely to interfere with resistance determinants before labor-intensive experimental testing (Stokes et al., 2020). These approaches are most effective when integrated with experimental validation, serving as hypothesis-generating tools rather than replacements for biochemical assays.

#### 7.3.2 Machine learning for structure–activity optimization

Machine-learning models trained on curated natural product datasets can identify structural features correlated with bioactivity, toxicity, or synergy. In recent studies, supervised learning has been used to optimize antibacterial and adjuvant properties by iteratively refining chemical structures based on experimental feedback (Ji et al., 2025; Chen Y. et al., 2024).

**Figure 4:**
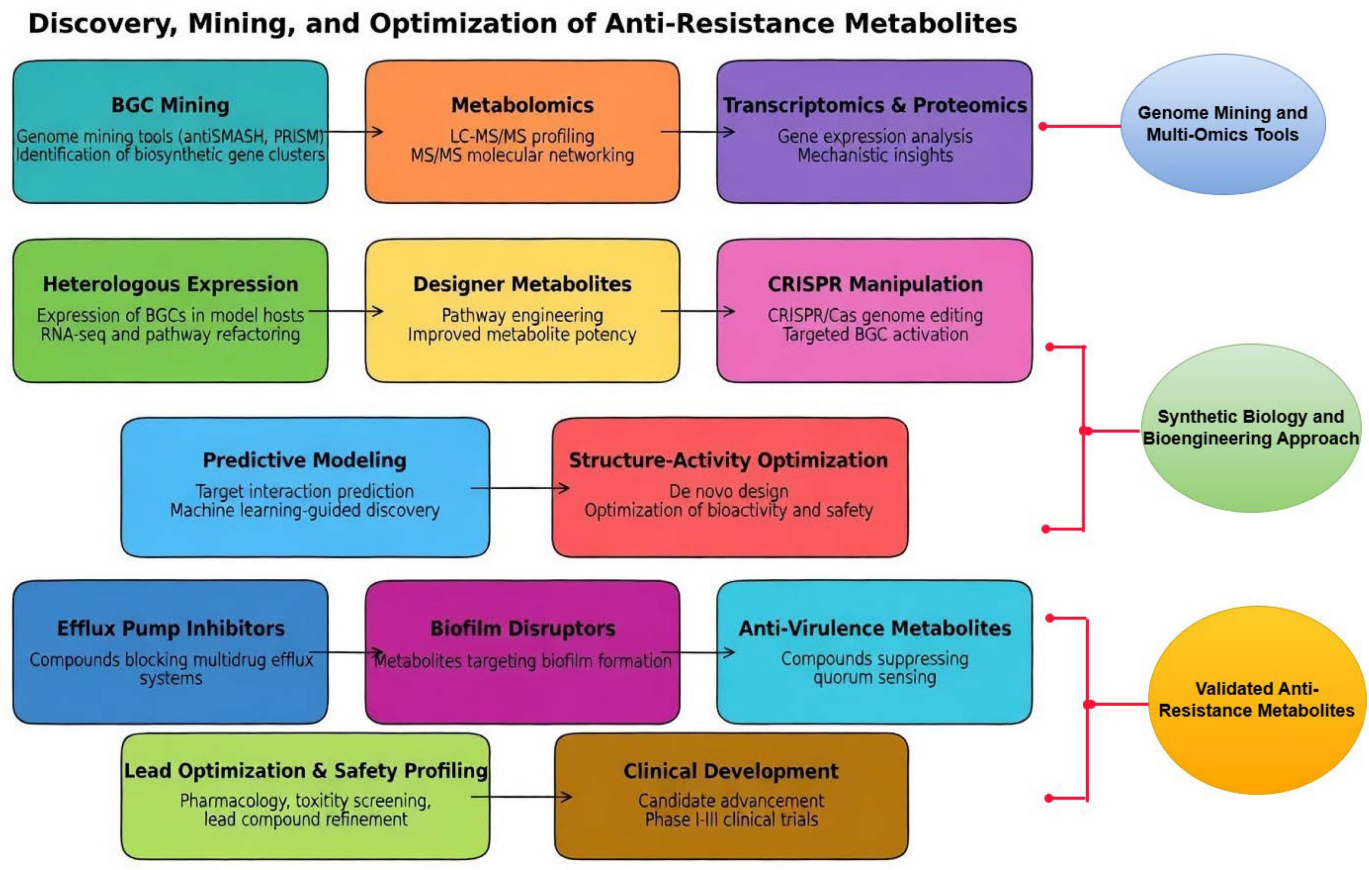
Discovery, mining, and optimization of anti-resistance metabolites. Integrated pipeline for the discovery and optimization of anti-resistance metabolites. The process begins with BGC mining using tools such as antiSMASH and PRISM, followed by metabolomics (LC–MS/MS profiling and molecular networking), transcriptomics/proteomics for mechanistic insights, and heterologous expression in model hosts. Subsequent steps include designer metabolite engineering, CRISPR-based BGC manipulation, machine learning-guided predictive modelling, structure–activity relationship (SAR) optimization, and development of efflux pump inhibitors, biofilm disruptors, and anti-virulence compounds, leading to lead refinement, safety profiling, and advancement to clinical phases.

For anti-resistance metabolites, ML-guided optimization is particularly promising for balancing efficacy, selectivity, and evolutionary robustness, enabling prioritization of compounds that potentiate antibiotics while minimizing selective pressure for resistance (see Figure 4).

## 8 Translational prospects and therapeutic development

While advances in genome mining, multi-omics, and synthetic biology have greatly expanded the repertoire of bacterial secondary metabolites with anti-resistance activity, their clinical impact ultimately depends on successful translation into safe, effective, and scalable therapeutics. Translating bacterial secondary metabolites from discovery into clinical anti-resistance therapeutics requires coordinated progress across pharmacology, formulation and delivery, preclinical validation, regulatory strategy, and manufacturing scale-up. Below, we synthesize current progress, principal challenges, and practical recommendations to accelerate candidate metabolites (and metabolite-based adjuvants) through the development pipeline.

### 8.1 Drug development pathways

#### 8.1.1 Pharmacodynamics and pharmacokinetic challenges

Natural products and metabolite-derived agents present unique PK/PD challenges that differ from small-molecule antibiotics. Complex structures (peptides, glycosylated macrolides, amphiphilic lipopeptides) often show limited oral bioavailability, rapid proteolytic degradation, and atypical tissue distribution; concurrently, their pharmacodynamics may be highly context dependent (e.g., potent biofilm disruption at concentrations that do not affect planktonic MIC) (Lombardi et al., 2025; Hajfathalian et al., 2024; Chen X. et al., 2022). These properties necessitate tailored PK/PD studies early in development to define exposure–response relationships relevant to their anti-resistance role (e.g., adjuvant vs. bactericidal agent). Comprehensive reviews synthesize and support integrating ADME profiling, stability testing, and mechanism-specific PD endpoints (biofilm MBEC, efflux inhibition assays, synergy indices) are essential to de-risk clinical translation (Simoben et al., 2023; Atanasov et al., 2021). Practical mitigation strategies include peptide stabilization (cyclization, D-amino acids), prodrug approaches, and formulation to extend half-life or target delivery. For adjuvants intended for co-administration with existing antibiotics, PK matching (synchronous exposure at the infection site) is critical—mismatched PK can eliminate synergy or promote resistance by exposing bacteria to sub-therapeutic drug levels. Recent studies on antibiotic adjuvants recommend early co-PK/PD studies to optimize dosing regimens (Dhanda et al., 2023).

#### 8.1.2 Stability and delivery system considerations

Delivery and stability are frequently the rate-limiting steps for natural products. Approaches that have matured in recent years include nanocarrier encapsulation (liposomes, polymeric nanoparticles), lipid- or peptide-based prodrugs, and targeted conjugates (e.g., siderophore–antibiotic conjugates) to improve permeability into Gram-negative pathogens (Padmanaban et al., 2025; Thakur et al., 2025). Nanoparticle platforms have shown promise for protecting labile metabolites from proteolysis, enhancing tissue targeting, and co-delivering metabolite–antibiotic pairs to infection foci (Roszkowski et al., 2025). Recent reviews summarize advances in nanoparticle formulations designed to overcome permeability and stability barriers for antimicrobial natural products and adjuvants (AlQurashi et al., 2025).

Design considerations should include: release kinetics compatible with the partner antibiotic; avoidance of carrier-induced toxicity or immune clearance; manufacturability and regulatory precedent; and the ability to scale. For metabolites intended to modulate host responses (immunomodulatory agents), targeted delivery to infection sites (e.g., inhalation for pulmonary infections, topical/coatings for device-associated infections) can both enhance efficacy and limit systemic exposure.

#### 8.1.3 Combination therapy design

Given that many secondary metabolites act as adjuvants (EPIs, *β*-lactamase suppressors, biofilm dispersers, QS inhibitors), intelligent combination design is central to translation. Key principles include mechanistic complementarity (e.g., pairing an efflux inhibitor with an efflux-exported antibiotic), PK/PD alignment, and use of checkerboard and time-kill assays, followed by *in vivo* combination efficacy and resistance-suppression studies. Modeling and empirical work indicate that well-designed combinations can produce synergistic bactericidal effects while reducing selective pressure for resistance, though careful dosing is required to avoid antagonism or pharmacokinetic mismatch (Roque-Borda et al., 2025; Wang B. et al., 2024). Consensus reviews of antibiotic adjuvants and combination therapies provide frameworks for dose-fractionation studies and translational progression (Kumar et al., 2023; Dhanda et al., 2023). Having outlined the key pharmacological, formulation, and combination-therapy considerations that govern early drug development, the discussion now turns to evidence supporting translational feasibility. Specifically, we examine preclinical and emerging clinical data demonstrating the efficacy of selected secondary metabolites and metabolite-based strategies in relevant infection models.

### 8.2 Preclinical and clinical advances

#### 8.2.1 Lead metabolites in development (2020–2025 pipeline)

Several promising leads and programs have advanced to preclinical validation between 2020 and 2025:

*Corramycin*, a novel peptidic natural product (antibiotic) isolated from *Corallococcus coralloides* (Renard et al., 2023), represents a rare example of a bacterial secondary metabolite with validated *in vivo* efficacy against Gram-negative pathogens. Mechanistically, corramycin enters bacterial cells via specific uptake pathways and disrupts essential cellular processes distinct from classical antibiotic targets, thereby avoiding cross-resistance with β-lactams and fluoroquinolones (Adam et al., 2024; Siebert and Kazmaier, 2024). In murine infection models, treatment resulted in significant reductions in bacterial burden, demonstrating that its intracellular mode of action translates into therapeutic efficacy rather than solely *in vitro* growth inhibition (Couturier et al., 2022).
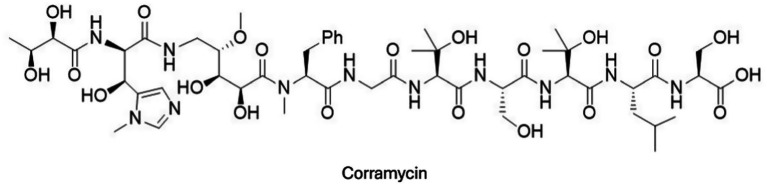


*Lasso peptides* (e.g., lariocidin-like RiPPs) show mechanistic promise due to their non-canonical ribosomal binding modes (Jangra et al., 2025). Structural and biochemical analyses demonstrate that these peptides bind the 30S ribosomal subunit at sites distinct from those targeted by aminoglycosides or tetracyclines, interfering with tRNA accommodation and translocation. This mechanism preserves activity against pathogens harboring ribosomal protection proteins or rRNA methylation, explaining their potency against carbapenem-resistant *Klebsiella pneumoniae*, *Acinetobacter baumannii*, and MDR *Escherichia coli*. The lasso topology further confers proteolytic stability, an important mechanistic advantage for systemic application (Jangra et al., 2025; Mucha et al., 2025).

*Natural efflux pump inhibitors (EPIs) and adjuvants* derived from microbial and plant-associated metabolites have progressed through mechanistic validation stages. These compounds inhibit RND-family efflux pumps either by direct occupation of substrate-binding pockets or by disrupting the proton motive force required for active extrusion, and biofilm dispersants in vitro and in small animal models (e.g., terpenoids, essential-oil components) (Thakur et al., 2025; Wiles et al., 2025; Rodrigues et al., 2020). Mechanistically, efflux inhibition has been quantified using ethidium bromide accumulation assays, showing increased intracellular drug retention, and checkerboard assays demonstrating 4–16-fold reductions in MICs of partner antibiotics. These data support the use of EPIs as resistance-modifying adjuvants rather than standalone antimicrobials. These candidates are often at the preclinical research stage, with translational advancement dependent on formulation and toxicity profiling (Gaurav et al., 2023; El-Demerdash et al., 2024). Collectively, these examples highlight a pipeline in which structurally novel natural products are transitioning from discovery to preclinical assessment, with heterogeneity in maturity and commercial interest.

#### 8.2.2 Animal model evidence for anti-resistance efficacy

Animal models have provided mechanistic confirmation that secondary metabolites can restore antibiotic efficacy by disabling resistance determinants *in vivo*.

In murine systemic and localized infection models, corramycin treatment reduced bacterial load and improved survival, correlating with its ability to evade efflux-mediated resistance and maintain intracellular accumulation. These findings link molecular uptake mechanisms directly to therapeutic outcomes.

In device-associated and biofilm infection models, biofilm-disrupting metabolites have shown enhanced bacterial clearance when combined with antibiotics (Taheri-Araghi, 2024; Ferris, 2021). Mechanistically, these metabolites degrade extracellular polymeric substances or inhibit matrix assembly, increasing antibiotic penetration and converting tolerant biofilm populations into susceptible states (Sychantha et al., 2021). Confocal microscopy and CFU quantification confirm reduced biofilm biomass and enhanced killing, linking structural biofilm disruption to therapeutic efficacy (Dhanda et al., 2023; Kumar et al., 2023). Collectively, these studies show that mechanistic targeting of resistance determinants (enzymes, efflux systems, biofilms)—rather than reliance on bactericidal pressure alone—can translate into meaningful *in vivo* outcomes.

### 8.3 Regulatory and manufacturing considerations

#### 8.3.1 Quality control and metabolite consistency: mechanistic implications

Natural products often present heterogeneity (mixtures of congeners, variable glycosylation, and stereochemistry), complicating quality control (QC). Regulatory authorities require well-characterized active ingredients, consistent manufacturing processes, and impurity profiling. Thus, regulatory approval of natural product–based therapeutics includes strict control over mechanistically relevant molecular features. For secondary metabolites, minor structural variations (e.g., side-chain length, stereochemistry, post-translational modifications) can significantly alter target binding, membrane interaction, or efflux susceptibility.

Mechanistically informed quality control, therefore, focuses on:

- *Defining the active molecular species* responsible for resistance modulation (e.g., the specific congener that inhibits efflux or binds the ribosome) (Klein-Junior et al., 2021). In addition to defining active molecular species, mechanistically informed quality control focuses on identifying the specific biological pathways and structural targets involved in resistance. Based on pharmacological and biochemical frameworks for 2025, these focuses include:
- *Target Interaction and Site Specificity:* Understanding how antibiotics or modulators manifest site-specific actions, such as binding to functional centers of the ribosome (e.g., the PTC or nascent peptide exit tunnel) to interfere with protein synthesis in a context-specific manner (Wilson et al., 2020; Krawczyk et al., 2024).
- *Identification of Resistance Modulators:* Focusing on genes and enzymes that chemically alter targets, such as the *erm* or *cfr* genes, which methylate 23S rRNA to reduce the binding affinity of various antibiotic classes (Yang et al., 2025; Kaitlyn Tsai et al., 2022).
- *Structural Barrier and Efflux Inhibition:* Characterizing mechanisms that directly obstruct protein exit ducts or collapse the energy gradients (e.g., proton gradients) necessary for multi-drug efflux pumps to function (Rouvier et al., 2025; Dey et al., 2022; Biswas et al., 2021).
- *Proteostasis and Cellular Fitness:* Analyzing how protein quality control (PQC) networks—including chaperones and proteases—maintain functional proteins and influence the evolutionary trajectory of antibiotic resistance mutations (Diaz Arenas et al., 2025; Ciccarelli, 2024; Godlewska et al., 2025).
- *Standardization of Natural Product Markers:* In pharmacognosy, applying compound-oriented approaches to ensure the identity and purity of plant-derived medicines by identifying biomarkers that correlate with therapeutic activity or resistance modulation (Amir et al., 2022; Klein-Junior et al., 2021).

*Analytical validation* using LC–MS/MS and NMR to ensure batch-to-batch consistency of structural features critical for activity (Gathungu et al., 2020). *Functional bioassays* (e.g., efflux inhibition, *β*-lactamase suppression, ribosomal binding) as release criteria, supplementing purely chemical specifications (Vinayagam et al., 2025).

For RiPPs and peptides, maintaining correct post-translational modifications and three-dimensional topology is essential, as alterations can abolish target engagement (Han and Won, 2024; Zhong et al., 2023). For adjuvant metabolites, preserving physicochemical properties that enable membrane partitioning or enzyme binding is similarly critical (Raghunath et al., 2024).

#### 8.3.2 Challenges for natural product commercialization

Despite their strong mechanistic rationale and promising preclinical performance, the successful commercialization of bacterial secondary metabolites faces substantial challenges arising from their chemical complexity, biosynthetic variability, and context-dependent efficacy. Addressing these barriers requires careful alignment of manufacturing strategies, regulatory pathways, and intellectual property frameworks with the unique biological and mechanistic attributes of natural product–based anti-resistance therapeutics.

Commercialization of secondary metabolites is constrained by several mechanistically rooted barriers. First, the scalability of production can conflict with biological activity: overexpression or pathway refactoring may alter folding, oxidation state, or congener ratios, leading to reduced affinity for resistance targets such as efflux pumps or metallo-β-lactamases (Han and Miao, 2024; Yasmeen, 2023). This necessitates iterative optimization of both biosynthetic pathways and downstream purification processes.

Second, mechanism-associated toxicity must be carefully managed. Metabolites that disrupt membranes, chelate metal ions, or modulate host immunity may exert off-target effects if systemic exposure is not tightly controlled (Richards et al., 2023; Zembroski and Buhman, 2020). Regulatory agencies, therefore, require comprehensive mechanistic toxicology studies, including target selectivity, host–pathogen discrimination, and safety margins relative to the intended resistance-modifying dose (Pognan et al., 2023; Muteeb et al., 2023; Kuppasani and Thomas, 2025).

Third, many secondary metabolites are most effective as antibiotic adjuvants, which suggests that their clinical benefit arises from mechanistic synergy rather than standalone antimicrobial activity. This creates regulatory complexity: efficacy must be demonstrated in combination with partner antibiotics, and clinical trial design must show restored susceptibility or improved outcomes relative to antibiotic monotherapy (VICH Steering Committee, 2024; Roque-Borda et al., 2025). Reviews of antimicrobial adjuvant development highlight that such combination-dependent mechanisms demand tailored regulatory pathways and biomarker-driven patient stratification (Dhanda et al., 2023; Kumar et al., 2023).

Finally, intellectual property (IP) protection for natural scaffolds remains challenging. As a result, successful commercialization strategies increasingly focus on engineered derivatives, optimized delivery systems, or defined combination regimens, rather than unmodified natural products.

- Engineered Derivatives: Creating novel, non-naturally occurring versions of the natural product allows for stronger patent protection (Hu et al., 2025; Ding and Xue, 2024). These modifications might involve changes to the chemical structure that enhance efficacy, reduce toxicity, or improve stability, making them distinct from the natural original.
- Optimized Delivery Systems: IP can be secured for the method of administering the compound, rather than the compound itself (Kumar Bandaru et al., 2021; Ding and Xue, 2024). This includes innovative formulations such as nanoparticles, specialized coatings, or targeted delivery mechanisms that improve the therapeutic outcome and qualify as a patentable invention.
- Defined Combination Regimens: Developing specific, non-obvious combinations of natural products with other compounds (either natural or synthetic) can also be patented. The novelty lies in the synergistic effect or improved treatment protocol achieved through the unique combination, rather than the individual components (Ding and Xue, 2024).

## 9 Challenges, knowledge gaps, and future directions

Translating bacterial secondary metabolites into broadly useful anti-resistance modalities requires confronting a set of scientific, methodological, and translational gaps. Below, we highlight the principal challenges, recommend pragmatic mitigation strategies, and identify priority research directions to accelerate safe, evolution-aware clinical deployment.

### 9.1 Resistance development risks and strategies to mitigate them

Resistance to any selective pressure is possible; therefore, deploying metabolites as therapeutics requires explicit assessment of evolutionary risk. Laboratory evolution experiments and comparative studies show that the rate and trajectories of resistance depend strongly on the mechanism of action (single-site vs. multimodal), exposure regimen, and ecological context (Yu et al., 2025; Maeda and Furusawa, 2024). For example, resistance evolves more rapidly to antibiotics with single, high-value molecular targets than to agents exerting multimodal membrane or metabolic stress, but compensatory evolution and horizontal gene transfer (HGT) can still enable persistence of resistance alleles in some settings. Experimental-evolution frameworks and integrated evolutionary analyses are essential to quantify these risks for each candidate metabolite (Woods et al., 2020; Kavya et al., 2023).

Mitigation strategies that have empirical or theoretical support include: *Use metabolites as adjuvants or anti-virulence agents* (lower direct lethality reduces selective sweeps for resistance) (Mahmud and Wakeman, 2024). *Design combination regimens* that create collateral sensitivity networks (pairing drugs such that resistance to one increase sensitivity to the other). Systematic mapping of collateral sensitivity can be used to select combinations that steer—not simply suppress—evolution Mahmud and Wakeman, 2024. *Limit environmental exposure* (avoid agricultural deployment that could disseminate selective pressure and mobilize resistance via environmental resistome). Environmental surveillance and stewardship frameworks should accompany any non-clinical use (Chen G. et al., 2022). *Perform experimental evolution under clinically relevant conditions* (biofilms, polymicrobial communities, host-mimicking media) to reveal realistic resistance trajectories rather than relying solely on planktonic assays (Jacobs et al., 2024; Maeda and Furusawa, 2024).

*Priority research*: standardized, publicly accessible pipelines for evolutionary risk assessment—combining experimental evolution, genomic surveillance, and fitness-cost modeling—should be developed and required during preclinical candidate evaluation.

### 9.2 Need for standardized assays for anti-resistance activity

Current antimicrobial screening paradigms (planktonic MIC) frequently fail to capture mechanistic outcomes relevant to anti-resistance modalities (e.g., efflux inhibition, biofilm dispersal, enzyme suppression). There is an urgent need for standardized assays and reporting frameworks tailored to anti-resistance activities:

- *Quantitative efflux assays* (ethidium bromide, Nile red accumulation with defined controls and pump-overexpressing reference strains) to measure functional EPI activity (Lu et al., 2020; Pal et al., 2020; Seukep et al., 2020).
- *Biofilm susceptibility testing* that goes beyond MIC to include MBEC (minimum biofilm eradication concentration), matrix integrity assays, live/dead confocal imaging, and standardized timepoints; recent critiques highlight heterogeneity in biofilm AST and propose harmonized methods (Coenye, 2023; Okae et al., 2022).
- *Enzyme inhibition assays* for *β*-lactamases and metallo-β-lactamases (e.g., kinetic IC₅₀, metal-chelation controls) with paired microbiological validation (restoration of β-lactam activity in isogenic strains) (Zhang S. et al., 2024; Hamrick et al., 2020; Ciuffreda et al., 2024).
- *Contextualized synergy testing* (checkerboard, time-kill curves, and *in vivo* combination studies) with explicit PK/PD alignment for both metabolite and antibiotic (Rodríguez-Gascón et al., 2021; Alikhani et al., 2025; Lorenzutti et al., 2021; Black et al., 2023). Establishing consensus protocols (analytical and biological) will improve comparability between labs, accelerate lead triage, and provide regulators with robust evidence of mechanism-specific activity.

### 9.3 Integrating ecological and evolutionary insights into drug design

Ecology and evolution offer actionable design principles: molecules evolved for multifunctionality, signaling disruption, or resource competition often exert mechanisms that are harder for pathogens to evade by simple point mutations. Translation requires intentionally integrating these insights:

- *Prioritize multimodal mechanisms* (membrane perturbation + efflux inhibition + quorum-sensing antagonism) to increase the mutational barrier to resistance. Experimental and theoretical work supports slower resistance emergence against multitarget agents (Yu et al., 2025; Maeda and Furusawa, 2024).
- *Exploit collateral sensitivity and trade-offs* by mapping evolutionary landscapes and designing sequential or cyclical regimens that select against durable resistance. Empirical collateral sensitivity mapping has matured and can inform rational pairings (Mahmud and Wakeman, 2024).
- *Use ecological mimicry in screening* (co-culture, nutrient limitation, host-derived matrices) to discover metabolites whose activity persists under realistic selective regimes, thereby reducing false positives that fail *in vivo* (Jacobs et al., 2024).

*Translational imperative:* incorporate ecological/evolutionary endpoints (resistance emergence rate, fitness costs, collateral sensitivity profiles) into go/no-go criteria for candidate advancement.

### 9.4 Promise of metabolite–nanocarrier systems

Nanocarriers can address key PK/PD and safety barriers for secondary metabolites while preserving mechanism-specific activities. Recent reviews and primary studies show nanocarrier approaches:

- *Protect labile metabolites* from proteolysis and metabolic clearance, extending circulation time and enabling lower systemic exposure (Lv et al., 2024; Antonelli and Palma, 2025).
- *Facilitate targeted delivery* (e.g., liposomal or polymeric carriers for pulmonary or topical applications) and co-delivery of metabolite–antibiotic pairs with synchronized release kinetics, which is critical for maintaining synergy and minimizing sub-therapeutic exposures that drive resistance (Khalid-Salako et al., 2025).
- *Modulate biodistribution* to concentrate adjuvants at infection sites (biofilms, intracellular niches) while reducing off-target host exposure and toxicity. Mechanistic studies document improved penetration into biofilms and enhanced antibiotic accumulation when paired with nanocarrier-delivered adjuvants (Lv et al., 2024; Antonelli and Palma, 2025).
- *Research priorities:* head-to-head comparisons of carrier platforms for specific mechanistic classes of metabolites (e.g., EPIs vs. ribosomal RiPPs), standardized assays for co-delivery kinetics, and robust toxicology focused on nanoparticle-metabolite interactions.

### 9.5 Opportunities for personalized medicine and microbiome-based therapeutics

Personalized medicine and microbiome-oriented therapeutics represent promising avenues to deploy metabolite-based anti-resistance strategies with improved efficacy and reduced evolutionary risk. Three complementary modalities deserve emphasis: (1) patient stratification using microbiome and resistance biomarkers to match mechanism-specific metabolites to the infection context; (2) direct therapeutic use of microbiome-derived metabolites (postbiotics) or engineered live biotherapeutics tailored to host and pathogen features; and (3) precision delivery systems and companion diagnostics that align pharmacokinetics, site of action, and mechanistic exposure. Below, we summarize mechanistic rationales, translational examples, and near-term research priorities.

#### 9.5.1 Biomarker-guided patient selection and companion diagnostics

Mechanistic matching between a metabolite’s mode of action and the patient’s pathogen/host biology increases the probability of therapeutic success. For example, identification of metallo-β-lactamase (MBL) genes in an isolate (e.g., bla < sub > NDM</sub>) would prioritize use of a metal-chelating MBL inhibitor as an adjuvant (Palacios et al., 2020; Guo et al., 2024); conversely, a strain whose resistance is dominated by over-expressed RND pumps would be a candidate for an efflux-inhibitor adjuvant (Thomson and Saadhali, 2025; Zhang S. et al., 2024; Zhang L. et al., 2024; Zahedi Bialvaei et al., 2021). Rapid molecular diagnostics and targeted sequencing of resistance determinants therefore provide the basis for companion diagnostic strategies that direct mechanism-matched metabolite use, minimizing off-target exposures and limiting selection pressure (Chigozie et al., 2025c). Some reviews emphasize microbiome and resistome profiling as actionable tools for precision therapy (Mousa, 2024; Allegretti et al., 2024).

Mechanistically, integrating pathogen genotyping with host microbiome signatures (e.g., dysbiosis patterns, presence/absence of key taxa that produce or metabolize candidate metabolites) can identify patients most likely to benefit from microbiome-derived adjuvants or postbiotic supplementation (Hasan et al., 2025). Multi-omics and machine-learning approaches that synthesize microbiome composition, metabolome readouts (e.g., SCFA and bile-acid profiles), and host immune markers are emerging as practical frameworks for stratification (Kumar et al., 2025).

#### 9.5.2 Postbiotics, metabolite therapeutics, and engineered microbiota

Direct therapeutic use of microbiome-derived metabolites (postbiotics)—such as short-chain fatty acids (SCFAs), specific bile-acid derivatives, or defined microbial peptides—offers advantages over live probiotics in terms of safety, stability, and standardization (Chigozie et al., 2025d). Mechanistically, postbiotics can modulate host immunity, strengthen barrier function, or perturb pathogen physiology (e.g., reduce virulence gene expression or biofilm formation), thereby acting as adjuncts that lower the antibiotic doses required and the selection intensity for resistance. Recent reviews synthesize evidence that SCFAs modulate inflammation and host defense, and that defined postbiotic preparations can be standardized for clinical use (Lange et al., 2023; Hijová, 2024).

Engineered live biotherapeutics—designer probiotic strains or consortia modified to produce specific anti-resistance metabolites (e.g., EPIs, quorum-sensing antagonists, or siderophore competitors)—provide sustained local metabolite release at infection sites (Iqbal et al., 2021; Ma et al., 2022). Mechanistically, such strains can outcompete pathogens for niches or deliver metabolites that disrupt resistance mechanisms *in situ*. Clinical translation of engineered microbiota requires strict biosafety designs (kill switches, auxotrophy) and robust regulatory strategies; reviews of precision microbiome therapeutics describe both the potential and the hurdles (Allegretti et al., 2024; Ahmed et al., 2025).

#### 9.5.3 Precision delivery and PK/PD alignment

For metabolite-based adjuvants, therapeutic benefit depends on achieving synchronized exposure with the partner antibiotic in the infection microenvironment. Nanocarrier systems, inhaled formulations (for pulmonary infections), or topical coatings (for device-associated infections) can concentrate metabolites where they are needed while minimizing systemic exposure and toxicity (Cojocaru et al., 2024; Cheng et al., 2025; Wang D. et al., 2024). Mechanistic studies show nanoparticle-mediated co-delivery can enhance antibiotic penetration into biofilms and maintain the local concentration ratios necessary for synergy (Makabenta et al., 2021; Joshi and Patil, 2025). Thus, development should target delivery platforms that preserve the metabolite’s mechanistic activity (e.g., avoid carriers that sequester active moieties) and permit controlled co-release (Amobonye et al., 2025; Jumaah et al., 2025).

#### 9.5.4 Microbiome-aware clinical trial designs

Clinical testing of metabolite interventions should incorporate microbiome and resistance biomarkers into eligibility criteria and endpoints. Examples of actionable trial design elements include: (a) stratifying enrollment by presence of target resistance determinants; (b) measuring fecal or tissue metabolome changes as pharmacodynamic biomarkers; (c) longitudinal resistome surveillance to monitor for emergent resistance; and (d) including host immune and microbiome recovery endpoints to capture broader benefits and risks. Emerging clinical reports on precision microbiome interventions (e.g., FMT and targeted probiotics) illustrate how such trial frameworks can reveal mechanistic efficacy signals and safety profiles (Goldiș et al., 2025; Allegretti et al., 2024).

#### 9.5.5 Research priorities

**Figure 5:**
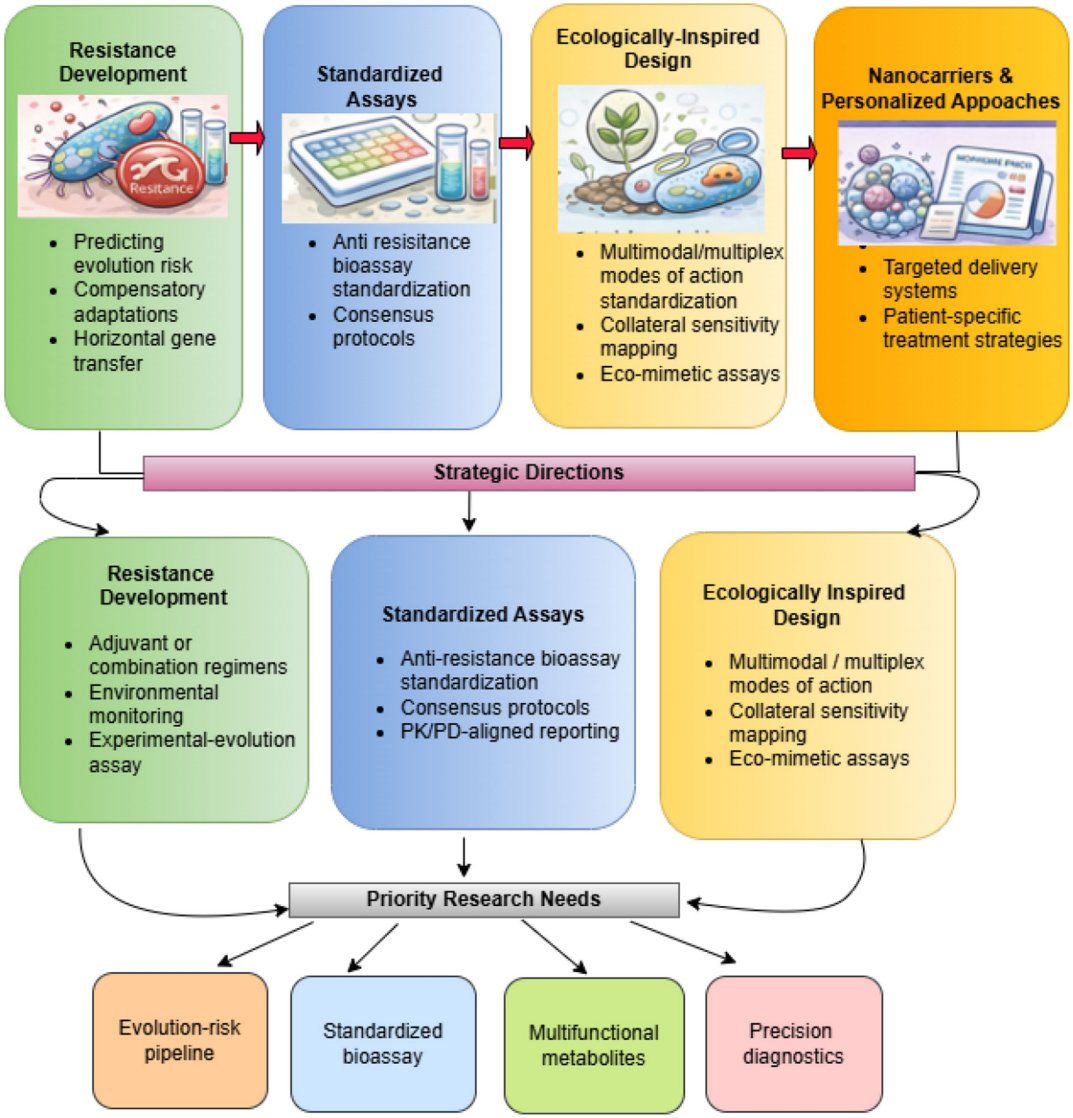
Challenges, knowledge gaps, and strategic directions for translating bacterial secondary metabolites as anti-resistance modalities. The figure summarizes key barriers to implementation, including resistance development risks, lack of standardized anti-resistance assays, and translational constraints, alongside corresponding mitigation strategies. Evolution-aware deployment, consensus bioassay frameworks, ecologically inspired multimodal design, and nanocarrier-enabled personalized approaches are highlighted as integrated priorities to guide safe, effective, and sustainable application of bacterial secondary metabolites in antimicrobial resistance management.

**Figure 6:**
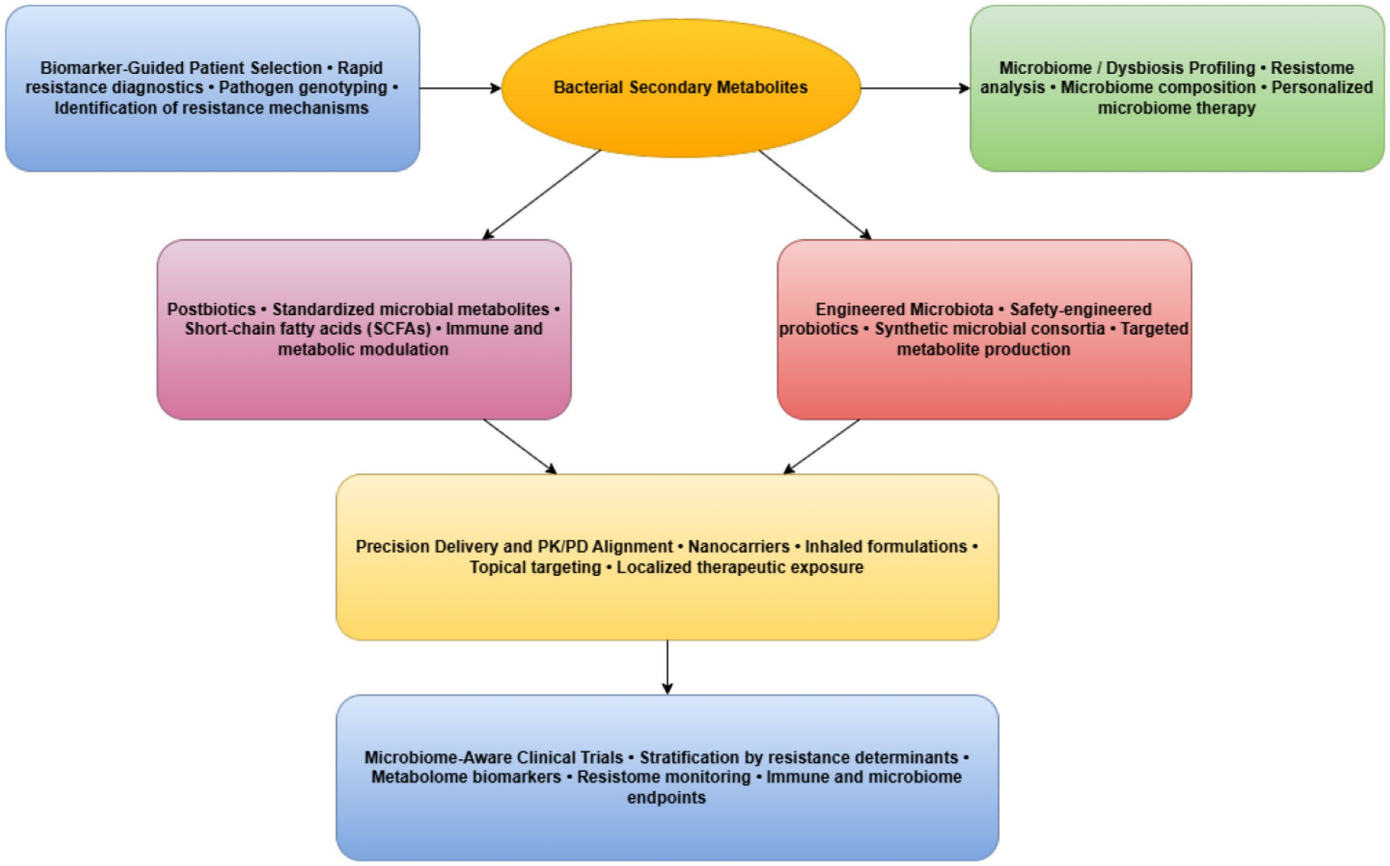
Precision and microbiome-informed strategies for deploying metabolite-based anti-resistance interventions. The figure illustrates biomarker-guided patient stratification, microbiome-derived therapeutics (postbiotics and engineered live biotherapeutics), and precision delivery systems that align pharmacokinetics and site-specific exposure with resistance mechanisms. Integration of pathogen genotyping, microbiome profiling, and microbiome-aware clinical trial designs enables targeted, evolution-aware application of bacterial secondary metabolites as anti-resistance modalities.

To operationalize personalized metabolite therapeutics, we recommend the following mechanistic research priorities:

1. Develop rapid companion diagnostics that detect resistance mechanisms and relevant microbiome features in clinically actionable timeframes.
2. Standardize postbiotic formulations and functional release assays that confirm retention of mechanism (e.g., efflux inhibition, quorum-sensing antagonism) after manufacture.
3. Advance engineered-microbe safety frameworks (genetic containment, functional insulation) and demonstrate *in vivo* mechanistic efficacy in relevant infection and colonization models.
4. Pursue PK/PD co-development of metabolite–antibiotic pairs, including targeted delivery platforms that permit synchronized microenvironmental exposure.
5. Embed longitudinal resistome and microbiome surveillance into early clinical studies to detect unforeseen selection pressures and guide stewardship (see Figures 5, 6).

## 10 Conclusion

Bacterial secondary metabolites represent a transformative and underexploited resource in the global effort to combat antimicrobial resistance (AMR). Beyond their historical role as sources of classical antibiotics, these molecules exhibit a broad spectrum of resistance-modifying activities, including efflux pump inhibition, suppression of resistance enzymes, disruption of biofilms and quorum sensing, ribosomal targeting through non-canonical binding modes, and immunomodulation. Collectively, these properties reposition secondary metabolites not merely as antimicrobials, but as anti-resistance modalities capable of restoring, potentiating, and prolonging the clinical utility of existing antibiotics.

Mechanistic and evolutionary insights strongly support this paradigm shift. Many secondary metabolites act through multimodal or context-dependent mechanisms that impose higher evolutionary barriers to resistance than single-target antibiotics. Their ecological origins—shaped by interspecies competition, signaling, and resource limitation—have endowed them with functionalities that destabilize resistance architectures and tolerance phenotypes rather than simply exert lethal pressure. When combined with principles from evolutionary biology, such as collateral sensitivity, fitness trade-offs, and ecological constraint, these mechanisms provide a rational foundation for designing therapies that are both effective and evolution-resilient.

Looking forward, accelerating the translation of bacterial secondary metabolites into clinically viable anti-resistance therapeutics will require an integrated roadmap. Key priorities include: (i) systematic discovery through genome mining, multi-omics, and AI-assisted approaches; (ii) rigorous mechanistic validation using standardized anti-resistance assays; (iii) optimization via synthetic biology, bioengineering, and advanced delivery systems, including nanocarriers; and (iv) translational strategies that incorporate PK/PD alignment, combination therapy design, and regulatory foresight. Equally important is the adoption of personalized and microbiome-aware frameworks, supported by companion diagnostics and evolutionary risk assessment, to ensure precise and responsible deployment.

In sum, bacterial secondary metabolites offer a scientifically robust and strategically flexible pathway to address AMR. By uniting mechanistic depth, evolutionary intelligence, and translational innovation, this class of molecules holds the potential to redefine how resistance is managed in the clinic—not by endlessly replacing failing antibiotics, but by systematically disarming the biological systems that sustain resistance itself.
